# Predicting kidney replacement therapy, cardiovascular disease and all-cause mortality in advanced chronic kidney disease among the Chinese population

**DOI:** 10.1080/0886022X.2025.2556301

**Published:** 2025-09-09

**Authors:** Xingchen Yao, Chenglong Li, Jinwei Wang, Lanlan Lu, Chao Yang, Luxia Zhang

**Affiliations:** ^a^ Renal Division, Department of Medicine, Peking University First Hospital, Peking University Institute of Nephrology, Beijing, China; ^b^ Research Units of Diagnosis and Treatment of Immune-Mediated Kidney Diseases, Chinese Academy of Medical Sciences, Beijing, China; ^c^ National Institute of Health Data Science at Peking University, Peking University Health Science Center, Beijing, China; ^d^ State Key Laboratory of Vascular Homeostasis and Remodeling, Peking University, Beijing, China; ^e^ Advanced Institute of Information Technology, Peking University, Hangzhou, China; ^f^ Xiaying Primary Health Care Center, Ningbo, China

**Keywords:** Advanced chronic kidney disease, external validation, kidney replacement therapy, cardiovascular disease

## Abstract

The Grams model, designed to predict adverse event risks in advanced chronic kidney disease (CKD) patients, was evaluated in a Chinese cohort of 1,333 patients with eGFR below 30 mL/min/1.73 m^2^. The model demonstrated moderate to good discrimination across outcomes, performing well in predicting kidney replacement therapy (KRT) but overestimating the risks of cardiovascular disease (CVD) and mortality. Calibration for KRT was accurate, while other outcomes required recalibration to improve alignment with observed data. Although recalibration enhanced calibration, it did not improve the model’s discrimination. Importantly, the study identified key predictors, such as albumin levels, that were not included in the original Grams model but significantly improved prognostic accuracy when incorporated. These findings suggest that while the Grams model has moderate applicability to the Chinese CKD population, its predictive performance can be improved by including additional critical variables. Further efforts are needed to improve the Grams model prior to application in the Chinese CKD population, such as the inclusion of novel valuable predictors.

## Introduction

Chronic kidney disease (CKD) poses a significant global health challenge, impacting approximately 10% of the worldwide population [1]. Patients with advanced CKD, characterized by an estimated glomerular filtration rate (eGFR) of ˂30 mL/min/1.73 m^2^, face a substantial burden of morbidity and an elevated risk of numerous adverse events [2]. Advanced CKD represents not only a high-risk subgroup within CKD but also a critical juncture in its management. For these patients, treatment priorities extend beyond merely preventing disease progression; they also encompass selecting appropriate dialysis methods, preparing for end-stage kidney disease (ESKD), and managing the complications associated with kidney disease [3]. To achieve these treatment goals, patients with advanced CKD need to have access to individualized, risk-based information [4]. Developing prognostic models can play a pivotal role in focusing efforts to prevent disease progression in advanced CKD and providing valuable insights for formulating treatment strategies [4].

Grams et al. [5] used 264,296 participants with eGFR < 30 mL/min/1.73 m^2^ from 29 cohorts in 30 countries to develop a prediction model that could estimate the two-year and four-year kidney replacement therapy (KRT), cardiovascular disease (CVD), and death simultaneously. This model shows a good prediction performance (concordance index [C-index]: 0.814 for two years and 0.929 for four years) in the developing cohorts. The model is named the Grams model. We selected the Grams model in our study because, first, compared to traditional models predicting the KRT events for patients with CKD, such as the kidney failure risk equation (KFRE) [6], the Grams model is the only one that was specially developed for patients with advanced CKD. In addition, previous validation studies have demonstrated that the prediction performance of the Grams model is better than that of the KFRE in patients with advanced CKD [7]. Second, traditional prediction models often overestimate the risk of KRT in patients with advanced CKD because they do not account for competing risks such as death [6]. The Grams model addresses this limitation by incorporating competing events into its predictions [5]. Third, compared to other models that focus solely on predicting KRT events, the Grams model is uniquely capable of predicting multiple outcomes, including KRT, CVD, and death within the same model. This is more in line with the complex clinical decision-making needs of patients with advanced CKD.

The Grams model has been validated in Western populations, with most research indicating that it demonstrates moderate to good discrimination and relatively accurate calibration [5,7–10]. However, no external validation has been conducted among the Chinese population, limiting its practical application and evaluation of clinical benefits in this group. Validation of the Grams model in the Chinese population is crucial due to significant variations in genetic, environmental, and lifestyle factors between populations, which can affect the model’s performance. Previous studies have shown that the prevalence of CVD risk factors differs between Chinese individuals and those in the United States. Additionally, research has reported that China lags behind developed countries in managing CVD risk factors [11]. These differences could impact the performance of the Grams model, underscoring the need to validate it for Chinese patients.

Previous studies have identified albumin, total cholesterol, high-density lipoprotein (HDL) cholesterol, hemoglobin, calcium, phosphorus, and bicarbonate as significant predictors for KRT, CVD, and death in patients with CKD [6,12–15]. However, these predictors were not included in the development of the Grams model. Evidence to determine whether incorporating these additional predictors could enhance the model’s performance in predicting adverse events in patients with advanced CKD is lacking.

Our study primarily aimed to: first, validate the Grams model using a multicenter, independent, and contemporary Chinese CKD cohort, and evaluate its potential clinical utility within this population. Second, we aimed to investigate whether there are additional important predictors, not included in the original Grams model, that can significantly enhance its performance in predicting the prognosis of patients with advanced CKD. This aspect of the study is intended to provide potential directions for further efforts aimed at improving the model.

## Methods

### Study population

Our participants were selected from the Chinese Cohort Study of Chronic Kidney Disease (C-STRIDE), a multicenter prospective longitudinal cohort study. This study involved 39 renal departments from various hospitals across 28 cities in 22 provinces in China (for further details, see ^Appendix^). The design and methods of the C-STRIDE have been previously described in detail [16]. The original cohort (*N* = 3,700) consisted of adults aged 18 to 74 years with CKD stages 1 to 4, enrolled between November 2011 and December 2016. For the present study, only participants with an eGFR <30 mL/min/1.73 m^2^ at baseline or observed during follow-up were included, resulting in a final sample size of 1,333. The participant selection process is illustrated in Figure S1. Importantly, no patients in the current cohort were included in the original Grams cohort. According to the Transparent Reporting of a Multivariable Model for Individual Prognosis or Diagnosis (TRIPOD) statements [17], the sample size was determined by the available data. Therefore, we did not justify the sample size of the dataset based on any *post hoc* sample size calculations.

### Outcomes

The primary outcomes of the study were: (1) initiation of KRT, (2) the first CVD event after eGFR falls below 30 mL/min/1.73 m^2^, and (3) all-cause mortality. Study outcomes were collected through telephone contact by research assistants or during routine outpatient visits every three to six months. Suspected outcomes were verified through medical records or death certificates. A committee of specialist physicians at Peking University First Hospital independently adjudicated the outcomes. KRT was defined as the commencement of maintenance hemodialysis, peritoneal dialysis, or kidney transplantation. CVD events included acute myocardial infarction, unstable angina, hospitalization for congestive heart failure, serious cardiac arrhythmias (such as resuscitated cardiac arrest, ventricular fibrillation, sustained or paroxysmal ventricular tachycardia, initial episodes of atrial fibrillation or flutter, severe bradycardia, or heart block), and cerebrovascular events [18,19]. The initial occurrence of any CVD event or initiation of KRT was designated as the index event. The time to any incident outcomes was calculated as the interval between baseline and the first occurrence of the event or censoring. For patients who progressed to eGFR < 30 mL/min/1.73 m^2^ during the follow-up period, the baseline date was defined as the date when eGFR < 30 mL/min/1.73m^2^ first occurred, and the follow-up time for these patients was calculated from this date. Follow-up continued until death, loss to follow-up, or December 31, 2017, whichever occurred first. Patients who were unreachable for more than six months were considered lost to follow-up.

### Predictors and risk estimation of the Grams model

The same predictors as those selected in the original Grams model were considered for this study. These included age, sex, race, history of CVD, smoking status, systolic blood pressure, diabetes mellitus, eGFR, and urine albumin-creatinine ratio (uACR). Age and race were self-reported. CVD history was defined as myocardial infarction, hospital admission for congestive heart failure, or severe cardiac arrhythmia incidents. This history was ascertained through self-reports from patients and a review of their medical records by trained staff during the baseline interview. Smoking status was defined as self-reported smoking history. Blood pressure was measured three times at 5-min intervals, and the mean value of the three readings was calculated. Diabetes was defined by individual cohorts as fasting glucose ≥7.0 mmol/L (126 mg/dL), hemoglobin A1c ≥6.5%, use of glucose-lowering drugs, or self-reported diabetes. Serum and urine creatinine levels were standardized to isotope dilution mass spectrometry centrally at Peking University First Hospital, and the uACR was calculated. Age, eGFR, and systolic blood pressure were center-scaled, and uACR was log-transformed and scaled to ln (10) before the analysis. The notable difference in the predictors’ definition compared to the original study was in smoking status; in the original study, it was defined as current smoking, whereas in our study, it was defined as smoking history. eGFR is a method used to estimate kidney function based on the blood creatinine test, age, gender, and race. eGFR can measure the level of kidney function and determine the stage of kidney disease. In this study, we used the Chronic Kidney Disease Epidemiology Collaboration (CKD-EPI) 2009 creatinine equation to calculate the eGFR [20]. The formula is as follows:

(1) $$\text{eGFR}=141\times {\text{min}(\text{Scr}/\kappa ,1)}^{\alpha }\times {\text{max}(\text{Scr}/\kappa ,1)}^{-1.209}\times 0.993^{\text{Age}}\times 1.018[\text{iffemale}]\times 1.159[\text{ifb}l\text{ack}]$$

Where *Scr* is serum creatinine, *κ* is 0.7 for females and 0.9 for males, and *α* is −0.329 for females and −0.411 for males.

According to the original model development study, the Grams model used a multinomial formula to predict the risk of KRT, CVD, and death, within two and four years. These outcomes are predicted in any possible sequence, including KRT only, KRT after CVD, CVD only, CVD after KRT, death after KRT, death after KRT and CVD, death after CVD and death only [5]. In addition, the multinomial coefficients information of the original Gram model was provided in the Supplemental material of the original literature [5]. To facilitate clinical application, our study consolidated these eight outcome events into three of the most common and significant clinical outcomes: KRT, CVD, and death.

### Clinical impact projection

To evaluate the clinical utility of the Grams model, we assessed whether decision rules based on predicted risk thresholds could identify patients who started KRT within one year, thereby being used to facilitate timely preparation for KRT initiation. Decision curve analysis (DCA) was used to evaluate and compare the net benefit of each decision rule, including thresholds for a two-year predicted KRT risk of 20%, 30%, 40%, and 50% based on the recalibrated Gram model. In addition, we evaluated eGFR thresholds of <30, <20, and <15 mL/min/1.73 m^2^, as well as the Kidney Foundation’s Kidney Disease Outcomes Quality Initiative suggested guideline of a two-year KRT risk >50% and/or an eGFR < 15mL/min/1.73 m^2^ [21]. Each patient was assessed whether their eGFR or KRT risk met or exceeded the specified thresholds. If patients reached the above thresholds, we observed whether they initiated KRT within one year; if so, the decision rule was ‘correct’ and KRT preparation was appropriate (a true positive). If the patients did not initiate KRT within one year, due to slow progression, death, or other causes, the KRT preparation was considered unnecessary or ‘incorrect’ at the time-point (a false positive) [7].

### Other predictors associated with adverse events in patients with advanced CKD

To explore the possibility of other important predictors not included in the Grams model, we selected additional variables based on previous classic literature and expert advice [6,12–15]. These variables include albumin, total cholesterol, HDL cholesterol, hemoglobin, serum calcium, serum phosphorus, and serum bicarbonate. All blood specimens were collected locally at each participating center and then transported *via* cold chain to the central laboratory of Peking University First Hospital after initial processing. Measurements of all serum biomarkers were conducted centrally at Peking University First Hospital.

### Statistical analysis

Continuous variables were summarized as mean ± standard deviation (SD) if normally distributed or median with the interquartile range (IQR) otherwise. While numbers (percentages) were reported for categorical variables. The observed occurrence of outcomes was described using crude incidence, person-year incidence rate, and four-year cumulative incidence. The cumulative incidence of KRT and CVD was estimated using the Cumulative Incidence Function (CIF), considering death as a competing event. The cumulative incidence of death was estimated using the Kaplan–Meier method.

We evaluated the performance of the Grams model using measures of discrimination and calibration. Discrimination, which refers to the model’s ability to correctly identify individuals who will or will not experience the outcome, was assessed using the time-to-event C-index. The R software package ‘QHScrnomo’ was employed to account for the competing risk of death. Calibration, which refers to the agreement between observed risk and absolute predicted risk, was assessed using calibration plots and the ratio of observed to expected outcomes (O/E ratio). Observed risk was estimated using the CIF accounting for the competing risk of death. Bootstrapping with 1,000 replications was used to generate 95% confidence intervals (CIs) for the C-index and O/E ratio. Clinical utility was assessed by DCA, a method that evaluates the association between the net benefit of the different clinical decision rules and threshold probabilities, and net benefit is plotted to evaluate the added value of different decision rules over a range of reasonable threshold probabilities. The calculation formula for the net benefit is as follows [22]:

(2) $$\text{NetBenefit}=\frac{\text{TruePositiveCount}}{n}-\frac{\text{FalsePositiveCount}}{n}(\frac{p_{t}}{1-p_{t}})$$

In this formula, *n* denotes the total number of patients, $p_{t}$ stands for the threshold probability. The threshold probability is the probability level at which a decision-maker chooses to intervene; if the probability exceeds this threshold, intervention is taken, and if it is below, no intervention is taken. In our study, the intervention referred to KRT preparation. The true-positive count referred to patients who experienced the event and were correctly recommended for intervention (predicted risk ≥ $p_{t}$), while the false-positive count referred to patients who did not experience the event but were unnecessarily recommended for intervention (predicted risk ≥ $p_{t}$). To assess whether decision rules had added value for clinical decision-making, the net benefit of decision rules was compared with two common decision rules in clinical practice: ‘treat all’ and ‘treat none’. The ‘treat all’ rule implied treating all patients, while the ‘treat none’ rule implied treating no patients. When comparing different decision rules, the rule with the highest net benefit across a range of threshold probabilities was considered most beneficial. The sensitivity, specificity, positive predictive value (PPV), and negative predictive value (NPV) were also calculated for each decision rule. Where the performance of the mode was suboptimal, recalibration was performed by adjusting the baseline risk of the Grams model. In our study, intercept recalibration was used to update the intercepts of eight linear predictors (LP) of the Grams model [23]:

(3) {LPnew,KRTonlyvsNoevents=α1+LPKRTonlyvsNoeventsLPnew,KRTafterCVDvsNoevents=α2+LPKRTafterCVDvsNoeventsLPnew,CVDonlyvsNoevents=α3+LPCVDonlyvsNoeventsLPnew,CVDafterKRTvsNoevents=α4+LPCVDafterKRTvsNoeventsLPnew,deathonlyvsNoevents=α5+LPdeathonlyvsNoeventsLPnew,deathafterKRTvsNoevents=α6+LPdeathafterKRTvsNoeventsLPnew,deathafterCVDvsNoevents=α7+LPdeathafterCVDvsNoeventsLPnew,deathafterKRT∧CVDvsNoevents=α8+LPdeathafterKRT∧CVDvsNoevents

Where $\alpha_{1},\alpha_{2},\ldots ,\alpha_{8}$ are the correction factors of intercept recalibration. They were calculated using the following formula [24]:

(4) $$\alpha \;=\;\text{ln}\left(\frac{\frac{\text{observed out}\;\text{come frequency}}{1-\text{observed out}\;\text{come frequency}}}{\frac{\text{mean predicted}\;\text{risk}}{1-\text{mean predicted}\;\text{risk}}}\right)$$

The R^2^ was used to evaluate the relative importance of the predictors included in the Grams model and other variables we selected. We developed six Cox regression models to predict the two-year and four-year risks of KRT, CVD, and death, with separate models for each outcome and time horizon. Each model incorporated two key components: (1) the predicted risk estimates from the original Grams model, which were time-aligned to the target prediction window (i.e., two-year Grams risk estimates for two-year models and four-year Grams risk estimates for four-year models), and (2) seven additional baseline laboratory variables (serum albumin, total cholesterol, HDL cholesterol, hemoglobin, calcium, phosphorus, and bicarbonate) to enhance predictive accuracy. Then, the time-dependent receiver operating characteristic (ROC) curves and the area under the receiver operating characteristic curve (AUC) were used to compare the accuracy of three new models with that of the Grams model. This analysis only included 1,119 patients with eGFR < 30mL/min/1.73m^2^ at baseline, considering the significant missing data of these new variables during the follow-up period.

Missing data were assumed to be missing randomly and were imputed using multiple imputation by chained equations with the R package ‘mice’. Fifteen imputed datasets were generated. Predictors requiring imputation included smoking status, uACR, and systolic blood pressure. The consistency of imputations was evaluated by comparing density plots and the model’s performance across each imputed dataset. Following the principle of optimal model performance, the dataset exhibiting the best performance was selected for the primary analysis.

We conducted several sensitivity analyses to ensure the robustness of our major findings. (1) To evaluate the impact of missing data and imputation, we performed both a complete case analysis and a per-imputation analysis of the 15 imputed datasets; (2) we further examined the model’s performance in different subpopulations (age, sex, and etiology subgroups) using calibration plots; (3) according to the original definition of smoking status in the Grams model, we reclassified smoking status as current smoking or not and repeated the main analysis.

The study adhered to the TRIPOD Statement for reporting (Shown in Supplemental Method). The statistical analysis was performed using Stata software (version 17.0; StataCorp LLC, Collage Station, TX, USA) and R software (version 4.2.2; R Foundation for Statistical Computing, Vienna, Austria). *p* Values <.05 were considered significant.

## Results

### Baseline characteristics

A total of 1,333 patients were enrolled in the study. At the first visit, where a participant had an observed eGFR <30 mL/min/1.73 m^2^, the mean age was 54 years (IQR, 43–64 years), and 699 (52.4%) were males, with no Black participants. The average eGFR was 22.3 mL/min/1.73 m^2^ (IQR, 17.3–26.3 mL/min/1.73 m^2^), and the mean uACR was 549.4 mg/g (IQR, 158.7–1,248.3 mg/g). Among all participants, 693 (52.0%) used antihypertensive medications, 217 (16.3%) used hypoglycemic medications, and 184 (13.8%) used a combination of both types of medications at baseline. A total of 390 (29.3%) participants used renin-angiotensin-aldosterone system inhibitors at baseline. Within two years, 238 (17.9%), 75 (5.6%), and 22 (1.7%) patients experienced KRT, CVD, and death, respectively. Within four years, 351 (26.3%), 104 (7.8%), and 62 (4.6%) patients experienced KRT, CVD, and death, respectively. The four-year cumulative incidence rates for KRT, CVD, and all-cause mortality were 28.3% (IQR, 25.8%–30.8%), 8.5% (IQR, 7.0%–10.1%), and 5.3% (IQR, 4.0%–6.6%), respectively. Additional baseline and follow-up characteristics are presented in Table S2.

Compared to the Grams cohort, participants in our cohort were younger (54 years [IQR, 43–64] vs. 72 years), had a lower history of CVD (13.1% vs. 45.1%), a lower prevalence of diabetes mellitus (26.4% vs. 46.2%), and a higher uACR (549.4 mg/g [IQR, 158.7–1,248.3] vs. 85 mg/g), as shown in Table S3. The median follow-up time for KRT, CVD, and death in our cohort was longer than that in the Grams cohort (3.8 years [IQR, 2.3–4.9] vs. 3.5 years, 4.3 years [IQR, 3.4–5.3] vs. 3.5 years, and 4.4 years [IQR, 3.6–5.4] vs. 3.5 years, respectively). The incidence of KRT was higher in our cohort than in the Grams cohort (386 [29.0%] vs. 31,541 [11.9%]), while the incidences of CVD and all-cause mortality were lower (118 [8.9%] vs. 70,394 [26.6%] and 88 [6.6%] vs. 123,985 [46.9%], respectively). The proportions of all outcomes are shown in Table S4. The missing rates of the predictors are presented in Table S5. Additionally, we compared the baseline and follow-up characteristics of the original data, imputed datasets, and complete cases, as shown in Table S6.

### Model discrimination and calibration

**Figure 1.**
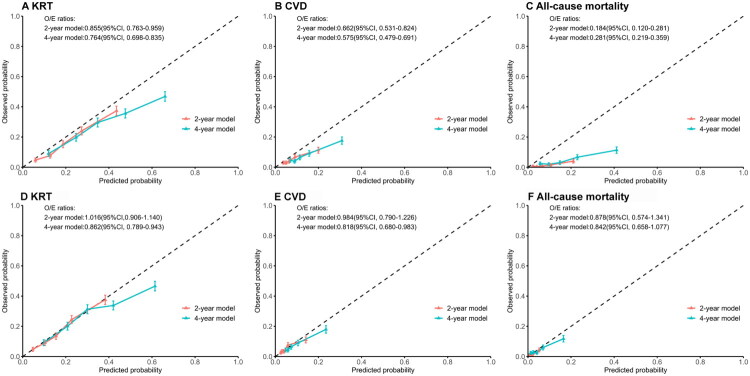
Calibration plots of the two-year and four-year Grams models before and after updating. The predicted probability is shown on the x-axis and the observed probability is given on the y-axis. The dotted 45°line represents perfect agreement between predicted and observed probability. The triangles represent a quintile of the validation population, ranked by predicted probability. Error bars represent 95% confidence intervals. Ratios of observed and expected outcomes were also been calculated to assess the calibration in large. (A) The calibration of predicting kidney replacement therapy before recalibration; (B) the calibration of predicting cardiovascular disease before recalibration; (C) the calibration of predicting death before recalibration; (D) the calibration of predicting kidney replacement therapy after recalibration; (E) the calibration of predicting cardiovascular disease after recalibration; and (F) the calibration of predicting death after recalibration. KRT: kidney replacement therapy; CVD: cardiovascular disease; O/E ratio: ratio of observed and expected outcomes; CI: confidence interval.

The Grams model demonstrated moderate to good discrimination for KRT, CVD, and death, with C-index values ranging from 0.656 to 0.720 (as shown in Table 1). Overall, the two-year Grams model exhibited better discrimination than the four-year Grams model. The discrimination for KRT and death was better than for CVD. Calibration plots and O/E ratios are presented in Figure 1. Overall, the model demonstrated relatively accurate calibration for KRT; however, for high-risk patients, it overestimated the risk of KRT. Additionally, the model overestimated the predicted probabilities of CVD and death, particularly the risk of death.

**Table 1.** Discrimination of the Grams model before and after updating.

Outcome	C-index (95% CI) before updating		C-index (95% CI) after updating	
	Two-year model	Four-year model	Two-year model	Four-year model
KRT	0.720 (0.688–0.752)	0.686 (0.659–0.714)	0.717 (0.686–0.747)	0.684 (0.658–0.712)
CVD	0.656 (0.593–0.721)	0.658 (0.601–0.710)	0.668 (0.605–0.731)	0.660 (0.605–0.714)
Death	0.712 (0.600–0.816)	0.684 (0.616–0.753)	0.688 (0.565–0.814)	0.677 (0.608–0.750)

Abbreviations: C-index: concordance index; CI: confidence interval; KRT: kidney replacement therapy; CVD: cardiovascular disease.

As shown in Figure 1, intercept recalibration led to closer alignment of observed and predicted risks. The ordering of participants’ predicted probabilities altered only slightly on recalibration. Hence, the C-index of the recalibrated model changed slightly. The C-index for predicting KRT, CVD, and death after recalibration ranged from 0.660 to 0.717 (shown in Table 1).

### Decision curve analysis

DCA of both the original and recalibrated models indicated potential clinical utility of the Grams model (as shown in Figure S2). The net benefit of using the Grams model to guide the clinical management decision was compared to the treat-all and treat-none strategies. The DCA curve was read vertically, meaning that for any given threshold probability, the decision curve with the best net benefit was the most beneficial for patients. At two years, basing clinical management decisions on predicted probabilities of KRT provided a benefit over the ‘treat-all’ and ‘treat-none’ strategies when using a treatment decision threshold probability of approximately 0.02 to 0.38 from the original model, or 0.02 to 0.34 from the recalibrated model. At four years, the Grams model also demonstrated a benefit over the ‘treat-all’ and ‘treat-none’ strategies when threshold probabilities were approximately 0.04 to 0.42 from the original model, or 0.07 to 0.46 from the recalibrated model.

Figure S3 shows the net benefit of using the Grams model and eGFR to guide KRT preparation. The results indicated that utilizing the Grams model to guide referrals was generally more effective than relying on an eGFR of less than 15 mL/min/1.73m^2^ when deciding about KRT preparation. When the threshold probability ranged from 0.06 to 0.16, the decision criterion of a two-year risk greater than 20% provided the best net benefit. Conversely, when the threshold probability ranged from 0.16 to 0.24, the decision criterion of a two-year risk greater than 30% yielded the best net benefit.

In addition, through the analysis of sensitivity, specificity (showed in Table S7), and other measures, we found that although the traditional eGFR < 15 strategy had a high specificity (84.6%), its sensitivity was extremely low (24.6%), resulting in as many as 75.4% of patients needing KRT being missed. Moreover, the PPV (13.0%) indicated that only 13% of referred patients actually needed KRT, highlighting the limitations of relying solely on the eGFR. In contrast, using the Grams model risk threshold (e.g., two-year risk >0.2) could increase sensitivity to 78.1% (95% CI: 69.4–85.3%), and the NPV (97.1%) further confirmed that 97% of non-referred patients did not need intervention, greatly reducing the risk of missed diagnoses. Although the model threshold >0.2 was accompanied by more false positives (392 vs. eGFR < 15′s 188), its number of false negatives (25) was only 29% of that with the eGFR < 15 strategy (86), significantly reducing the risk of clinical deterioration due to delayed referrals. Additionally, the model threshold >0.3 had a specificity (86.1%) similar to eGFR < 15, while its sensitivity (45.6%) was approximately twice as high, providing an ideal balance point for resource-limited scenarios. Notably, the combined strategy (two-year KRT risk >0.5 and/or eGFR < 15) did not show synergistic value, suggesting that patients with eGFR < 15 were already a high-risk group, and the model’s stratification effect was limited.

### Newly identified predictors of study outcomes

**Figure 2.**
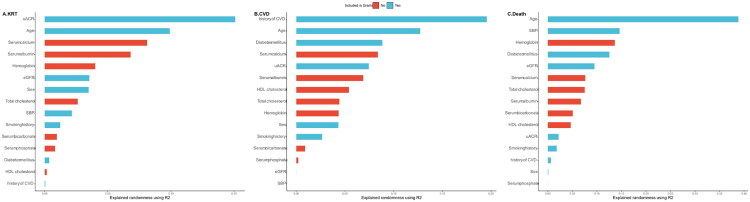
Relative importance of the predictors in the Grams model and other predictors. X-axis represents the relative importance of the predictors using R^2^, Y-axis represents all predictors. The length of the bar represents the importance of the predictors. Red bars represent predictors not included in the Grams model and blue bars represent the predictors in the Grams model. (A) Relative importance of the predictors for KRT; (B) relative importance of the predictors for CVD; and (C) relative importance of the predictors for death. KRT: kidney replacement therapy; CVD: cardiovascular disease.

**Figure 3.**
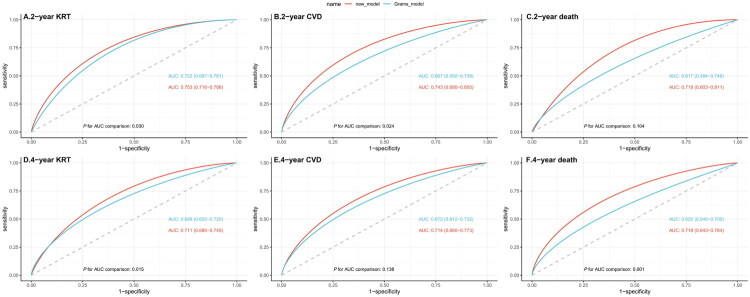
Receiver operation characteristic analysis comparing the performance of new models and Grams model. (A) ROC curves for two-year KRT; (B) ROC curves for two-year CVD; (C) ROC curves for two-year death; (D) ROC curves for four-year KRT; (E) ROC curves for four-year CVD; and (F) ROC curves for four-year death. ROC: receiver operation characteristic; KRT: kidney replacement therapy; CVD: cardiovascular disease.

According to the result of R^2^ (shown in Figure 2), new variables such as albumin, total cholesterol, HDL cholesterol, hemoglobin, serum calcium, serum phosphorus, and serum bicarbonate were important for predicting the risks of KRT, CVD, and death. However, these variables were not included in the Grams model, which may contribute to its suboptimal performance in the Chinese population. The ROC curves illustrated this option preliminarily. Compared with the original Grams model, six new models using predictors that included the risk of KRT, CVD and death estimated by original Grams model and these new variables (albumin, total cholesterol, HDL cholesterol, hemoglobin, serum calcium, serum phosphorus, and serum bicarbonate) showed higher AUC ranging from 0.711 to 0.753 for predicting the KRT, CVD and death separately (shown in Figure 3).

### Sensitivity analysis

As shown in Tables S8 and S9, Figures S4 and S5, the Grams model’s prediction performance was not materially altered across complete cases (*N* = 909) and 15 imputed datasets. The performance of subgroups was visually explored by calibration plots as shown in Figures S6–S11, and the results were similar to the main analysis. In addition, recategorizing the smoking status as current smoking or not did not change significantly (shown in Table S10, Figure S12).

## Discussion

In this multicenter validation study of Chinese patients with advanced CKD, we assessed the performance of the Grams model in predicting the risks of KRT, CVD, and death within two and four years. The model demonstrated moderately effective predictive performance for three outcomes. Incorporating additional predictors that were overlooked by the Grams model could potentially enhance its predictive capabilities. To the best of our knowledge, this is the first study to externally validate the Grams model in the Chinese population with advanced CKD.

Several validation studies conducted within Western populations have been published, and their results exhibited some heterogeneity compared to our findings. The original development study reported that the prediction performance for KRT was good [5]. Ramspek et al. [7] Thanabalasingam et al. [9] and Prouvot et al. [8,25] demonstrated that the model exhibited modest to good discrimination for two-year and four-year KRT (C-index: 0.64–0.82), CVD (C-index: 0.69–0.70), and death (C-index: 0.61–0.70) in three different Western populations. These findings are consistent with our results. Regarding calibration, the Grams model, after recalibration, also showed relatively accurate calibration.

In summary, we found that the discrimination of the Grams model was suboptimal in almost all external validation cohorts. According to our results, the unsatisfactory discrimination of the Grams model might be due to the lack of important predictors. Tangri et al. identified serum albumin, calcium, bicarbonate, and phosphate among numerous candidate variables as being associated with the risk of kidney failure, which could enhance the predictive ability of eGFR and albuminuria for kidney failure [6]. Cholesterol is a well-recognized risk factor for CVD [26], and has been widely incorporated into risk estimation tools, such as Pooled Cohort Equations [13] and Systematic Coronary Risk Estimation 2 [14]. However, these critical risk factors were all overlooked by the Grams model. According to the R^2^ results, these predictors are also important for estimating the risk of KRT, CVD, and death in patients with advanced CKD. The ROC curves and AUC of the new models incorporating these predictors were significantly higher than those of the Grams model. This suggests that the suboptimal performance of the Grams model may be due to the omission of important predictors, highlighting potential areas for further improvement of the model in patients with advanced CKD in China.

However, the main reason for the less-than-ideal calibration of the Grams model in our study compared to the original development study might be caused by the outcome incidence. The incidence of CVD and death in our cohort was comparatively lower than that in the original Grams model, which resulted in the model obviously overestimating the risk of CVD and death in our cohort. The lower incidence of CVD and death in our cohort can be due to several possible reasons. First, participants’ characteristics differed [27]. Compared with the original cohort, patients in our cohort were younger (54 [IQR, 43–64] years vs. 72 years). Previous studies have demonstrated that younger age was associated with a higher risk of KRT and a lower risk of CVD and death [19,28]. Additionally, our population had a lower prevalence of CVD history (13% vs. 45.1%), which could help explain the lower incidence of CVD events and death according to previous studies [28]. Moreover, in our cohort, the proportion of glomerulonephritis was higher than diabetic nephropathy. Prior investigations have revealed that compared to diabetic nephropathy, the incidence of CVD and death was lower in patients with glomerulonephritis [29,30]. Second, despite the standardized quality controlling procedures regarding outcomes reporting and reviewing in our study, event underreporting was not completely prevented, leading to the potential underestimation of event incidence. To improve the calibration of the model, we performed intercept calibration, which significantly enhanced the model’s calibration performance.

Although predictive accuracy at the individual level was moderate, the DCA showed that the Grams model could provide net benefits across an extensive range of threshold probabilities. Our analysis suggested that we could selectively intervene for patients with advanced CKD, using a risk-based approach. For example, the Grams model could potentially be used to determine the optimal timing for KRT preparation, such as the placement of an arteriovenous fistula (AVF), as suggested by a report from the Kidney Disease Improving Global Outcomes group [4]. The referral and placement of AVF currently mainly rely on the physician’s expertise, which may lead to the placement of AVF either too early or too late, both of which are detrimental [31]. Implementing a prediction model for optimal timing can reduce the number of patients undergoing unnecessary vascular access surgery and increase the likelihood of initiating dialysis with a mature AVF. Except for the preparation of placement of AVF, the Grams model can also be used to guide medication in patients with advanced CKD. For instance, it enables the targeted administration of cardiorenal protective medications to high-risk patients, thereby maximizing the efficacy of the treatment while preventing overmedication in low-risk patients. Moreover, the Grams model can be instrumental in the screening and selection of participants for clinical trials. By accurately identifying and selecting suitable trial participants, the model can help accelerate the clinical trial process.

This study reveals a significant paradigm shift in clinical decision-making: moving from reliance on a single eGFR threshold to risk assessment based on multidimensional models to guide KRT preparation. Compared to the single eGFR threshold, the Grams model demonstrates superior net benefits within clinically relevant probability thresholds, consistent with evidence from other studies [7]. This finding is particularly important for elderly patients, who often exhibit heterogeneity in disease progression at the same eGFR levels. By quantifying individualized two-year risks, the model enables clinicians to achieve two key objectives: prioritizing early intervention for rapid progressors (e.g., those with high uACR or comorbidities) and avoiding overtreatment of slow progressors – a balance that cannot be achieved by relying solely on eGFR. The model’s flexibility is further highlighted by its threshold-dependent characteristics: low-risk thresholds (>20%) are suitable for scenarios where delayed KRT could have severe consequences (e.g., younger patients), maximizing sensitivity to avoid missed diagnoses; whereas high-risk thresholds (>30%) control false positives, meeting the needs of resource-limited settings. Notably, the ineffectiveness of the combined strategy (eGFR < 15 combined with two-year KRT risk >50%) suggests that for patients who have already entered advanced CKD (eGFR < 15), the necessity of routine KRT preparation outweighs the value of further risk stratification. These findings challenge the traditional approach of using a single eGFR for clinical decision making, advocating for the establishment of individualized decision-making pathways based on risk stratification.

This study had several strengths. First, we demonstrated that some important variables overlooked by the Grams model are also crucial for predicting the prognosis of patients with advanced CKD. Incorporating these variables could enhance the performance of the prediction model. Second, we accounted for censoring and competing risks in the external validation to ensure that the predicted risk was not overestimated. This is particularly important for elderly patients and individuals with multiple long-term conditions, where an overestimation of the predicted risk might contribute to overtreatment. Third, the use of data from a current, multicenter cohort allowed for the generalization of our results to various regions of China. Fourth, the size of the current cohort allowed for the identification of more than 200 KRT events, a figure suggested to be required to ensure consistency of results in external validation [32].

## Limitations

Our study also had important limitations. First, the study population was exclusively derived from the nephrology departments of tertiary hospitals, where patients received standardized CKD management. This limits the generalizability of the findings to the broader Chinese CKD population. Previous studies have shown that the awareness rate of CKD among Chinese patients is only about 10% [33,34]. Our team’s prior analysis based on regional healthcare data revealed that among patients with CKD who underwent Scr testing, 61.4% missed any CKD-related diagnoses, and only 2.9% received accurate CKD-staging diagnoses, and merely 7.1% of general hospitals had nephrologists [35]. These data collectively reveal that in China, CKD awareness is low, diagnosis and management are suboptimal, and nephrology resources are scarce. This situation leads to a significant disparity in disease progression and outcomes: compared to patients with CKD receiving standardized management in nephrology departments of tertiary hospitals, the most patients with CKD who do not receive specialized care face accelerated disease progression, with higher risks of progressing to ESKD, as well as increased risks of CVD and mortality. Consequently, the discrimination and calibration of the Grams model will differ when applied to patients who do not receive standardized care in tertiary hospital nephrology departments, compared to those who do. Second, in the original study, smoking status was defined as current smoking; however, due to missing data in our cohort, we used smoking history for the main analysis. Although this might affect the model’s performance, we expected the impact to be minimal. This was confirmed by repeating the primary analysis in 691 patients with known current smoking status, yielding similar results. Third, despite establishing quality control measures for outcomes reporting and reviewing, underreported events could not be entirely precluded, potentially leading to an underestimation of event incidence. Fourth, although the size of our cohort enabled the identification of over 200 KRT events, the incidence of the other two outcomes (CVD and death) was relatively low. This might impact the consistency of the results associated with these two outcomes and also prevent us from performing more complex model recalibrations, such as re-estimating regression coefficients or adding additional predictors [23]. Finally, missing data also presented a challenge for our study. Although we used multiple imputation – a robust method to handle missing data – it may introduce bias when the data are not randomly missing [36]. However, the bias introduced by this method is not greater than that associated with complete case analysis, and it permitted a larger sample size, thereby increasing statistical power [36].

In summary, we externally validated the Grams model in a multicenter, independent, and contemporary Chinese cohort. The Grams model exhibited moderate performance in the Chinese population. Additionally, some important predictors had not been included in the Grams model. These findings suggest the need for further efforts to improve the Grams model before applying it to the Chinese CKD population, such as incorporating novel and valuable predictors.

## Supplementary Material

SUPPLEMENTAL MATERIAL_RF.pdf

supplemental_figures.zip

## Data Availability

The datasets generated during and/or analyzed during the current study are available from the corresponding author on reasonable request.

## Generative artificial intelligence (AI)

Because we wrote the manuscript in a non-native language, we used ChatGPT (version GPT-4o) for language improvement.

## Appendix

### C-STRIDE collaborators

Peking University First Hospital: Ming‐Hui Zhao and Luxia Zhang; the Affiliated Hospital of Hubei Traditional Chinese Medical College: Xiaoqin Wang and Jun Yuan; the Xiangya Hospital of Central South University: Qiaoling Zhou and Qiongjing Yuan; General Hospital of Ningxia Medical University: Menghua Chen and Xiaoling Zhou; the Second Hospital of Hebei Medical University: Shuxia Fu and Shaomei Li; Guizhou Provincial People’s Hospital: Yan Zha and Rongsai Huang; the First Affiliated Hospital of Zhengzhou University: Zhangsuo Liu and Jun Zhang; Sichuan Academy of Medical Sciences and Sichuan Provincial People’s Hospital: Li Wang and Lei Pu; the First Affiliated Hospital of Xinjiang University of Medicine: Jian Liu and Suhua Li; Peking University Shenzhen Hospital: Zuying Xiong and Wei Liang; Xinqiao Hospital: Jinghong Zhao and Jiao Mu; the Second Affiliated Hospital of Kunming Medical College: Xiyan Lian and Yunjuan Liao; the First Affiliated Hospital of Chongqing University of Medicine: Hua Gan and Liping Liao; Shandong Provincial Hospital: Rong Wang and Zhimei Lv; the First Affiliated Hospital of Guangxi University of Medicine: Yunhua Liao and Ling Pan; the First Affiliated Hospital of the Medical College, Shihezi University: Xiaoping Yang and Zhifeng Lin; Yuxi City People’s Hospital: Zongwu Tong and Yun Zhu; Beilun People’s Hospital in Ningbo: Qiang He and Fuquan Wu; the Second Affiliated Hospital of Tianjin University of Medicine: Rong Li and Kai Rong; the First Affiliated Hospital of Baotou Medical College: Caili Wang and Yanhui Zhang; Peking University Third Hospital: Yue Wang and Wen Tang; Beijing Hospital of Ministry of Health: Hua Wu and Ban Zhao; the Second Hospital of Shanxi University of Medicine: Rongshan Li and Lihua Wang; Shengjing Hospital of China Medical University: Detian Li and Feng Du; the First Affiliated Hospital of Anhui University of Medicine: Yonggui Wu and Wei Zhang; Tianjin Medical University General Hospital: Shan Lin and Pengcheng Xu; the First Affiliated Hospital of Dalian University of Medicine: Hongli Lin; Shandong University Qilu Hospital: Zhao Hu and Fei Pei; the Affiliated Hospital of Hebei University: Haisong Zhang and Yan Gao; Dongzhimen Hospital Affiliated to Beijing University of Chinese Medicine: Luying Sun and Xia Li; Chifeng Second Hospital: Wenke Wang and Fengling Lv; the Second Affiliated Hospital of Anhui University of Medicine: Deguang Wang and Xuerong Wang; Qianfoshan Hospital: Dongmei Xu and Lijun Tang; China Rehabilitation Research Center, Beijing Boai Hospital: Yingchun Ma and Tingting Wang; West China Hospital of Sichuan University: Ping Fu and Tingli Wang; the First Affiliated Hospital with Nanjing Medical University: Changying Xing and Chengning Zhang; Minhang Central Hospital: Xudong Xu and Haidong He; the Second Affiliated Hospital of Chongqing University of Medicine: Xiaohui Liao and Shuqin Xie; and the Affiliated Hospital of Chengde Medical College: Guicai Hu and Lan Huang.
