# Supplementary material for: Predicting kidney replacement therapy, cardiovascular disease and all-cause mortality in advanced chronic kidney disease among the Chinese population
Source: Ren Fail. 2025 Sep 9;47(1):2556301. doi: 10.1080/0886022X.2025.2556301 (PMC12422041; doi:10.1080/0886022X.2025.2556301)
Supplement: SUPPLEMENTAL MATERIAL_RF.pdf [file IRNF_A_2556301_SM3110.pdf]

SUPPLEMENTAL MATERIAL

Table of contents

TRIPOD Checklist 3

Supplemental Methods 8

Supplemental Figures 9

Supplemental Tables 9

TRIPOD Checklist

Section/Topic		Checklist Item	Section/Paragraph
**Title and abstract**			
Title	1	Identify the study as developing and/or validating a multivariable prediction model, the target population, and the outcome to be predicted.	Title
Abstract	2	Provide a summary of objectives, study design, setting, participants, sample size, predictors, outcome, statistical analysis, results, and conclusions.	Abstract
**Introduction**			
Background and objectives	3a	Explain the medical context (including whether diagnostic or prognostic) and rationale for developing or validating the multivariable prediction model, including references to existing models.	Introduction, Para 1-3
	3b	Specify the objectives, including whether the study describes the development or validation of the model or both.	Introduction, Para 5
**Methods**			

Source of data	4a	Describe the study design or source of data (e.g., randomized trial, cohort, or registry data), separately for the development and validation data sets, if applicable.	Methods, Para 1
	4b	Specify the key study dates, including start of accrual; end of accrual; and, if applicable, end of follow-up.	Methods, Para 1-2
Participants	5a	Specify key elements of the study setting (e.g., primary care, secondary care, general population) including number and location of centers.	Methods, Para 1
	5b	Describe eligibility criteria for participants.	Methods, Para 1
	5c	Give details of treatments received, if relevant.	n/a
Outcome	6a	Clearly define the outcome that is predicted by the prediction model, including how and when assessed.	Methods, Para 2
	6b	Report any actions to blind assessment of the outcome to be predicted.	n/a
Predictors	7a	Clearly define all predictors used in developing or validating the multivariable prediction model, including how and when they were measured.	Methods, Para 3
	7b	Report any actions to blind assessment of predictors for the outcome and other	n/a

		predictors.	
Sample size	8	Explain how the study size was arrived at.	Methods, Para 1
Missing data	9	Describe how missing data were handled (e.g., complete-case analysis, single imputation, multiple imputation) with details of any imputation method.	Methods, Para 12
Statistical analysis methods	10c	For validation, describe how the predictions were calculated.	Methods, Para 4, Supplemental material
	10d	Specify all measures used to assess model performance and, if relevant, to compare multiple models.	Methods Para 8-9
	10e	Describe any model updating (e.g., recalibration) arising from the validation, if done.	Methods Para 9-10
Risk groups	11	Provide details on how risk groups were created, if done.	n/a
Development vs. validation	12	For validation, identify any differences from the development data in setting, eligibility criteria, outcome, and predictors.	Methods, Para 3
**Results**			
Participants	13a	Describe the flow of participants through the study, including the number of	Results, Para 1

		participants with and without the outcome and, if applicable, a summary of the follow-up time. A diagram may be helpful.	Supplemental material
	13b	Describe the characteristics of the participants (basic demographics, clinical features, available predictors), including the number of participants with missing data for predictors and outcome.	Results, Para 1<br>Supplemental material
	13c	For validation, show a comparison with the development data of the distribution of important variables (demographics, predictors and outcome).	Results, Para 2,<br>Supplemental material
Model performance	16	Report performance measures (with CIs) for the prediction model.	Results, Para 3-6 & Table 1<br>&Figure 1
Model-updating	17	If done, report the results from any model updating (i.e., model specification, model performance).	Results, Para 4 & Table 1<br>& Figure 1<br>Supplemental material
**Discussion**			
Limitations	18	Discuss any limitations of the study (such as nonrepresentative sample, few events per	Discussion, Para 8

		predictor, missing data).	
Interpretation	19a	For validation, discuss the results with reference to performance in the development data, and any other validation data.	Discussion, Para 2
	19b	Give an overall interpretation of the results, considering objectives, limitations, results from similar studies, and other relevant evidence.	Discussion, Para 1
Implications	20	Discuss the potential clinical use of the model and implications for future research.	Discussion, Para 5-6
**Other information**			
Supplementary information	21	Provide information about the availability of supplementary resources, such as study protocol, Web calculator, and data sets.	n/a
Funding	22	Give the source of funding and the role of the funders for the present study.	Submission information

Supplemental Methods

Predicted risk calculations

Firstly, we calculated the probabilities of 8 outcomes (kidney replacement therapy (KRT) only, KRT after cardiovascular disease (CVD), CVD only, CVD after KRT, death after KRT, death after KRT and CVD, death after CVD and death only). The detailed methodology could be found in the Grams model development research[1]. And the multinomial coefficients were provided in the Grams supplementary material Table S7[1]. Variables were entered as follows: age was centered at 60 years and entered per 10 years; systolic blood pressure was centered at 140mmHg and entered per 20 mmHg; estimated glomerular filtration rate (eGFR) was centered at 25 ml/min/1.73m^2 and entered per 5 ml/min/1.73m^2; for sex, male was entered as 1, female as 0; for race, black was entered as 1, rest as 0; for diabetes, CVD history and smoking status, yes is entered as 1, no as 0; urine albumin-to-creatinine ratio (uACR), was log-transformed and scaled to ln(10). Here is the detailed mathematical and steps used:

1. Linear Regression Model:

The basic form of a linear regression model is:

$$Y = \beta_0 + \beta_1 X_1 + \beta_2 X_2 + \ldots + \beta_n X_n$$

Where Y is the outcome variable, $X_1, X_2, \ldots, X_n$ are the predictor variables, and $\beta_0, \beta_1, \ldots, \beta_n$ are the coefficients.

2. Probability Calculation for 8 outcome events:

Probabilities for each outcome are calculated using a multinomial logistic regression model:

$$P(Y_i) = \frac{\exp(X\beta_1)}{1 + \sum_{j=1}^{k} \exp(X\beta_j)}$$

Where $P(Y_i)$ is the probability of the i-th outcome, X is the matrix containing all variables and their interaction terms, and β_i is the corresponding coefficient vector.

3. Probability Calculation for KRT, CVD and death:

We calculated the probabilities of three main outcomes (KRT, CVD and death) by combining specific probabilities. This method has been used in the previous study[2].

$$P_{KRT} = P_{KRT\ only} + P_{KRT\ after\ CVD} + P_{CVD\ after\ KRT} + P_{death\ after\ KRT} + P_{death\ after\ KRT\ and\ CVD}$$

$$P_{CVD} = P_{CVD\ only} + P_{KRT\ after\ CVD} + P_{CVD\ after\ KRT} + P_{death\ after\ CVD} + P_{death\ after\ KRT\ and\ CVD}$$

$$P_{death} = P_{death\ after\ KRT} + P_{death\ after\ CVD} + P_{death\ after\ KRT\ and\ CVD} + P_{death\ only}$$

Supplemental Figures

Figure S1. Participants selection diagram.

Figure S2. Decision curves for the 2-year and 4-year Grams model before and after u pdating.

Figure S3. Decision curves using decision criteria incorporating the prediction risks of the Grams model, eGFR thresholds, and the combined diagnostic criteria of eGFR and predicted risk.

Figure S4. Calibration plots of the 2-year and 4-year Grams model in all imputed data sets.

Figure S5. Calibration plots of the 2-year and 4-year Grams model in complete cases (N=909).

Figure S6. Calibration plots of the 2-year and 4-year Grams model among age<65 ye ars patients(N=1014).

Figure S7. Calibration plots of the 2-year and 4-year Grams model among age≥65 ye ars patients(N=319).

Figure S8. Calibration plots of the 2-year and 4-year Grams model among male pati ents(N=698).

Figure S9. Calibration plots of the 2-year and 4-year Grams model among female pa tients(N=634).

Figure S10. Calibration plots of the 2-year and 4-year Grams model among glomerul onephritis patients(N=519).

Figure S11. Calibration plots of the 2-year and 4-year Grams model among diabetic k idney disease patients(N=216).

Figure S12. Calibration plots of the 2-year and 4-year Grams model when smoking st atus was defined as current smoking (N=691).

Supplemental Tables

Table S1. Correction factors of intercept recalibration.

Table S2. Baseline and Follow-up characteristics of study participants

Table S3. Baseline and follow-up characteristics, compared to the development cohort.

Table S4. The proportions of all outcomes.

Table S5. Missing rates of predictors.

Table S6. Comparison of baseline and follow-up characteristics of original data, multi ple imputed datasets, and complete cases.

Table S7. Comparison of diagnostic performance for timing of KRT preparation based on different eGFR and Grams model risk thresholds.

Table S8. Discrimination of Grams model in all imputed datasets, 2(a) and 4(b) years.

Table S9. Discrimination of the Grams model in complete cases(N=909).

Table S10. Discrimination of the Grams model when smoking status was defined as cu rrent smoking (N=691).

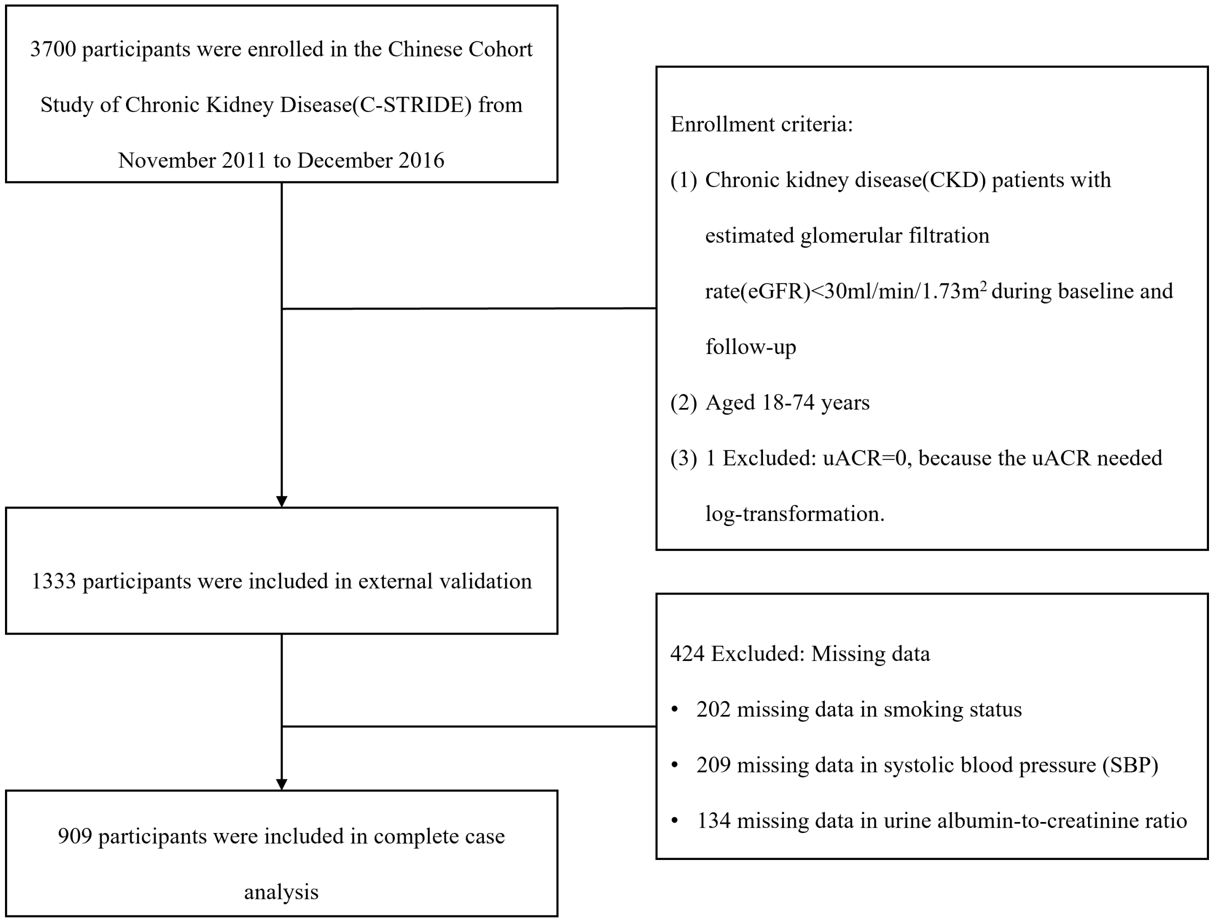

Figure S1. Participants selection diagram.

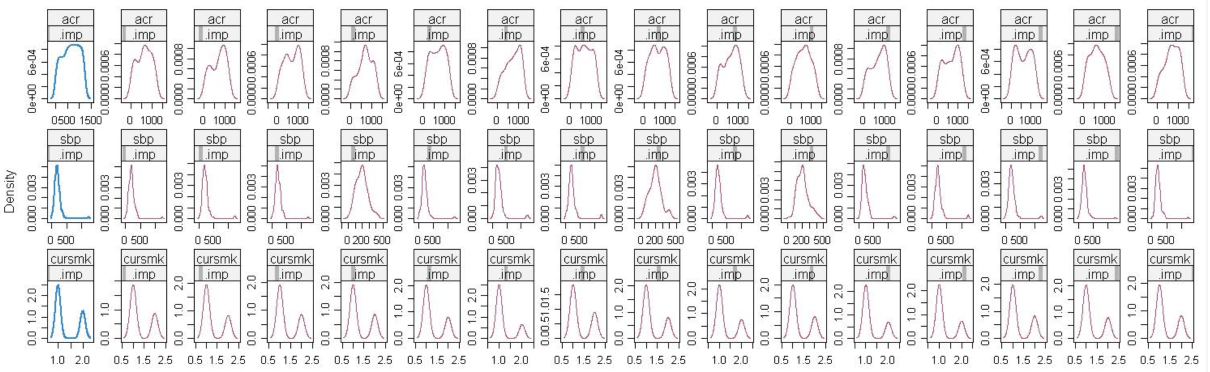

Figure S2. Density plots of original data and 15 imputed datasets. The three rows represent three predictors that need to be imputed: urine albumin-to-creatinine ratio (uACR), systolic blood pressure and smoking status. Blue lines indicate original data and red lines indicate imputed datasets.

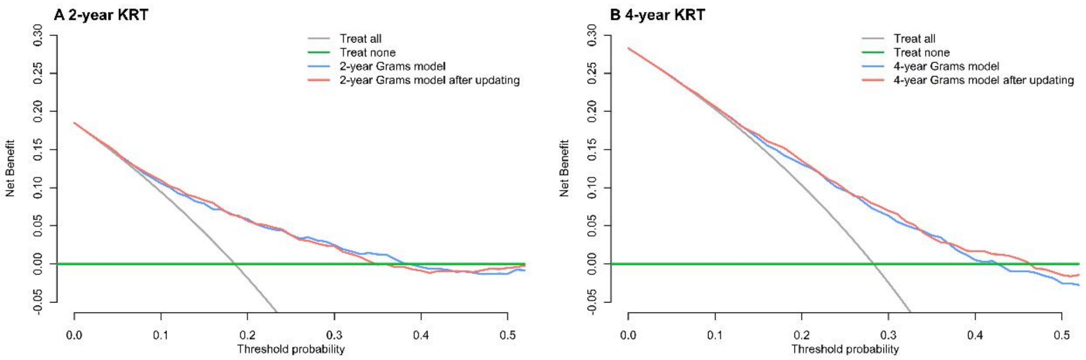

Figure S2. Decision curves for the 2-year and 4-year Grams model before and after updating. X-axis represents a range of threshold probabilities; y-axis represents the net benefit for given threshold probabilities. The blue and red curves depict the net benefit of the Grams model before and after updating.

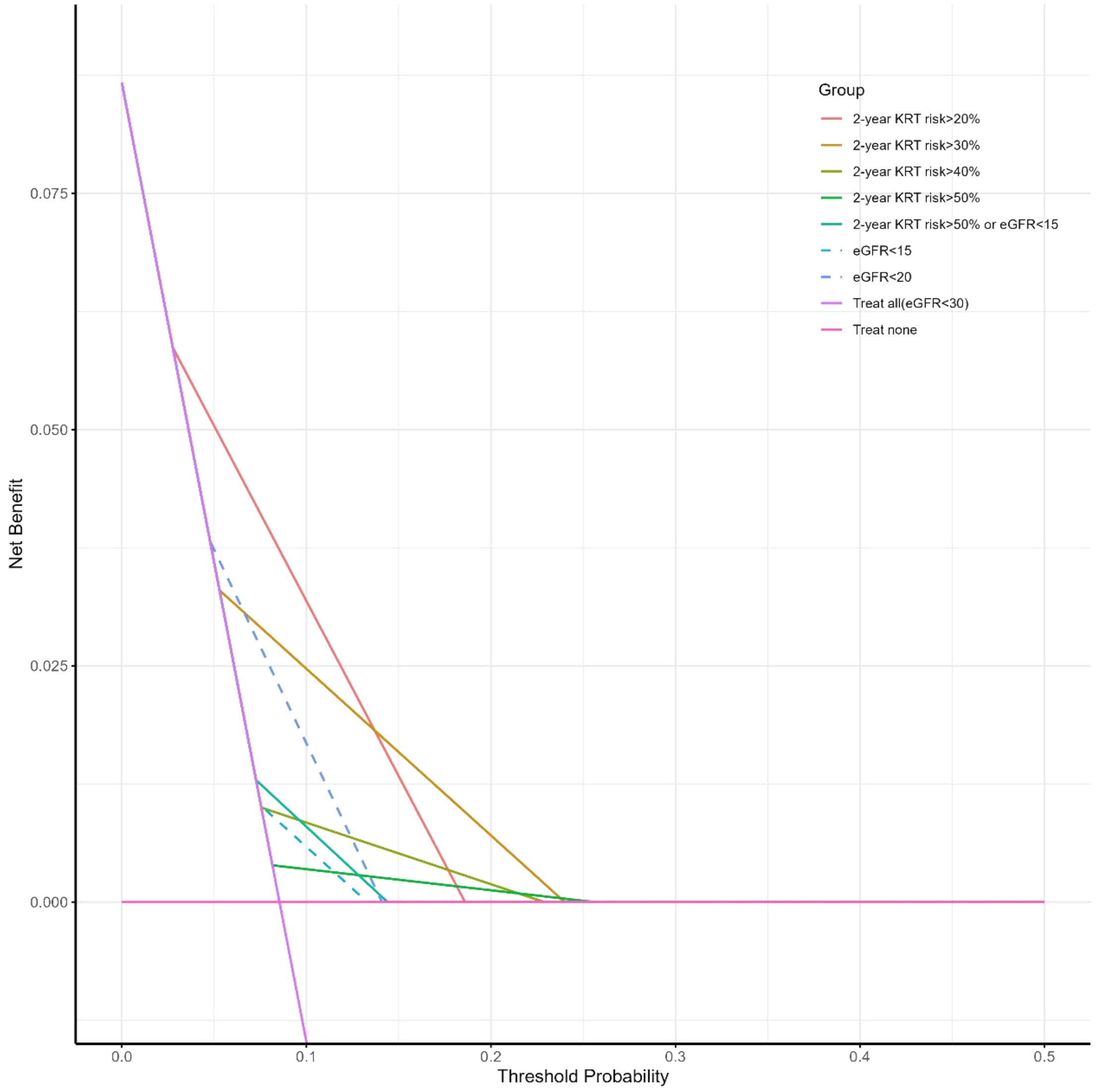

Figure S3**. Decision curves using decision criteria incorporating the prediction risks of the Grams model, eGFR thresholds, and the combined diagnostic criteria of eGFR and predicted risk.** X-axis represents a range of threshold probabilities; y-axis represents the net benefit for given threshold probabilities. Different colored lines represent various decision criteria. KRT, kidney replacement therapy; eGFR, estimated glomerular filtration rate.

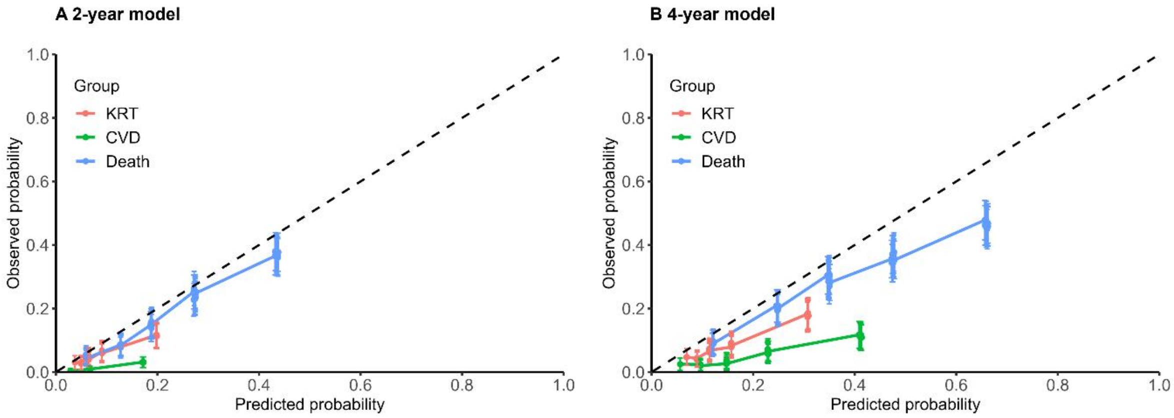

Figure S4. Calibration plots of the 2-year and 4-year Grams model in all imputed datasets. The predicted probability is shown on the x-axis and the observed probability is given on the y-axis. The dotted 45-degree line represents perfect agreement between predicted and observed probability. The dots represent a quintile of the validation population, ranked by predicted probability. Error bars represent 95% confidence intervals. (A), the calibration plots for 2-year Grams model; (B), the calibration plots for 4-year Grams model. KRT, kidney replacement therapy; CVD, cardiovascular disease.

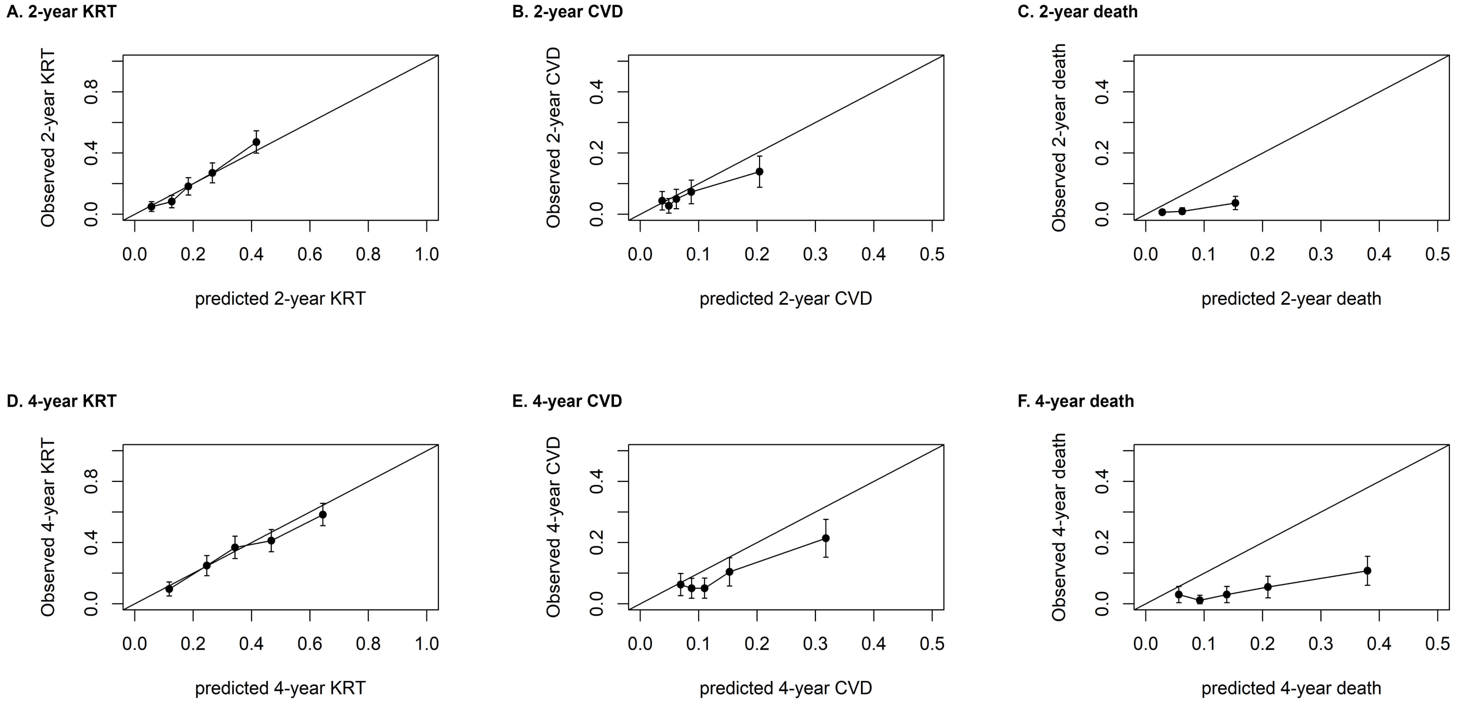

Figure S5. Calibration plots of the 2-year and 4-year Grams model in complete cases (N=909). The predicted probability is shown on the x-axis and the observed probability is given on the y-axis. The dotted 45-degree line represents perfect agreement between predicted and observed probability. The dots represent a quintile of the validation population, ranked by predicted probability. Error bars represent 95% confidence intervals. (A), the calibration plot for 2-year KRT; (B), the calibration plot for 2-year CVD; (C), the calibration plot for 2-year death; (D), the calibration for 4-year KRT; (E), the calibration plot for 4-year CVD; (F), the calibration plot for 4-year death. KRT, kidney replacement therapy; CVD, cardiovascular disease.

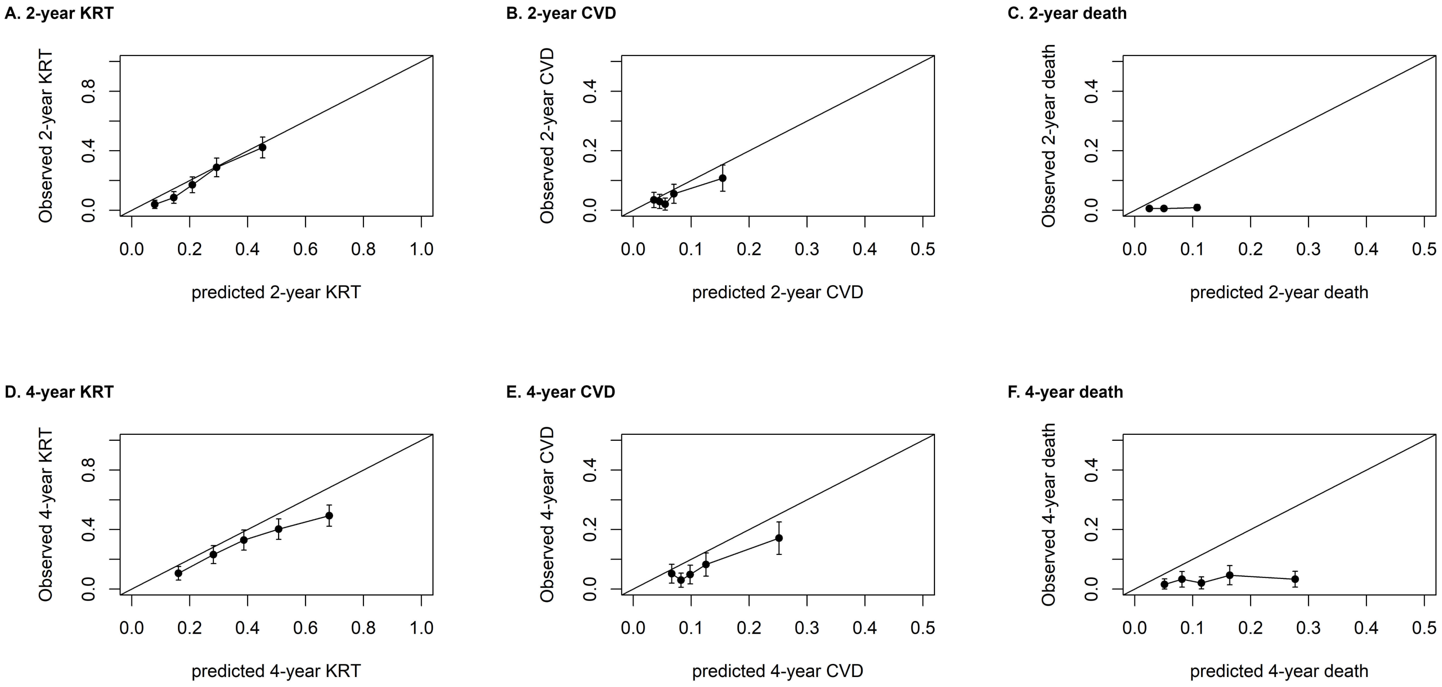

Figure S6. Calibration plots of the 2-year and 4-year Grams model among age<65 years patients (N=1014). The predicted probability is shown on the x-axis and the observed probability is given on the y-axis. The dotted 45-degree line represents perfect agreement between predicted and observed probability. Error bars represent 95% confidence intervals. (A), the calibration plot for 2-year KRT; (B), the calibration plot for 2-year CVD; (C), the calibration plot for 2-year death; (D), the calibration for 4-year KRT; (E), the calibration plot for 4-year CVD; (F), the calibration plot for 4-year death. KRT, kidney replacement therapy; CVD, cardiovascular disease.

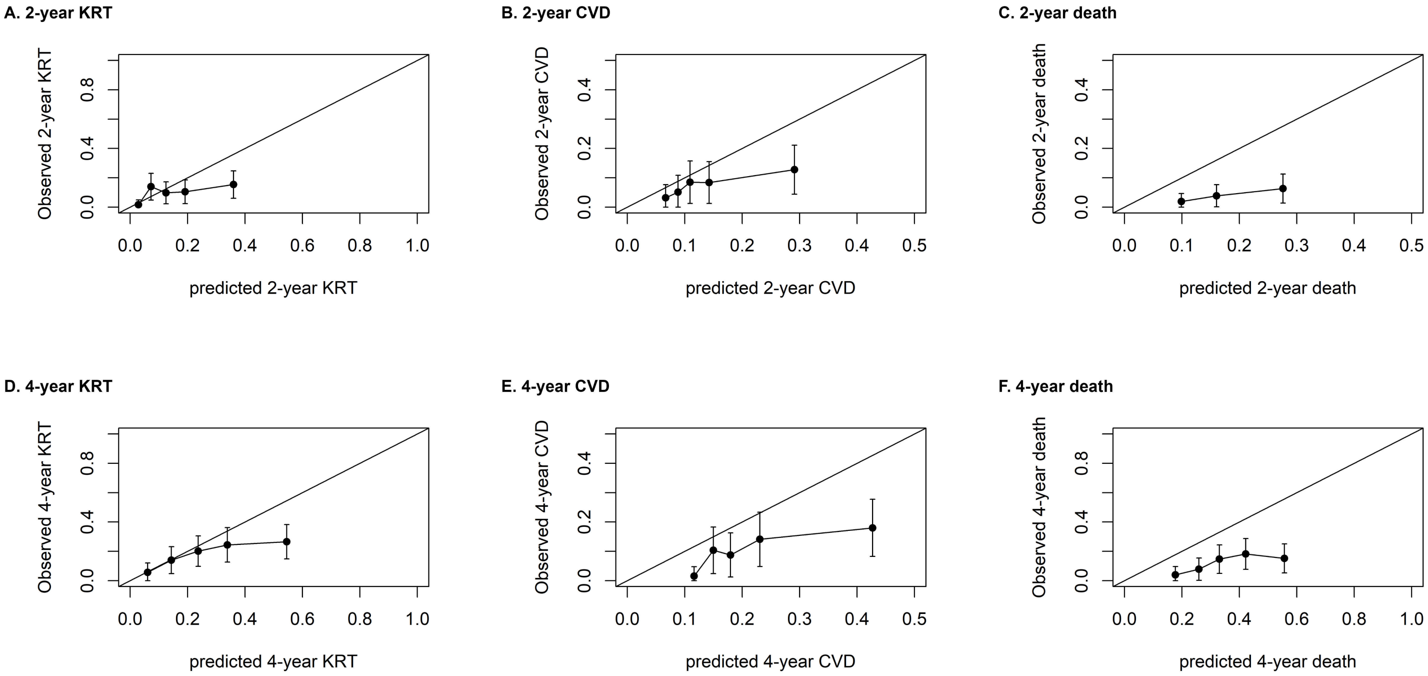

Figure S7. Calibration plots of the 2-year and 4-year Grams model among age≥65 years patients (N=319). The predicted probability is shown on the x-axis and the observed probability is given on the y-axis. The dotted 45-degree line represents perfect agreement between predicted and observed probability. Error bars represent 95% confidence intervals. (A), the calibration plot for 2-year KRT; (B), the calibration plot for 2-year CVD; (C), the calibration plot for 2-year death; (D), the calibration for 4-year KRT; (E), the calibration plot for 4-year CVD; (F), the calibration plot for 4-year death. KRT, kidney replacement therapy; CVD, cardiovascular disease.

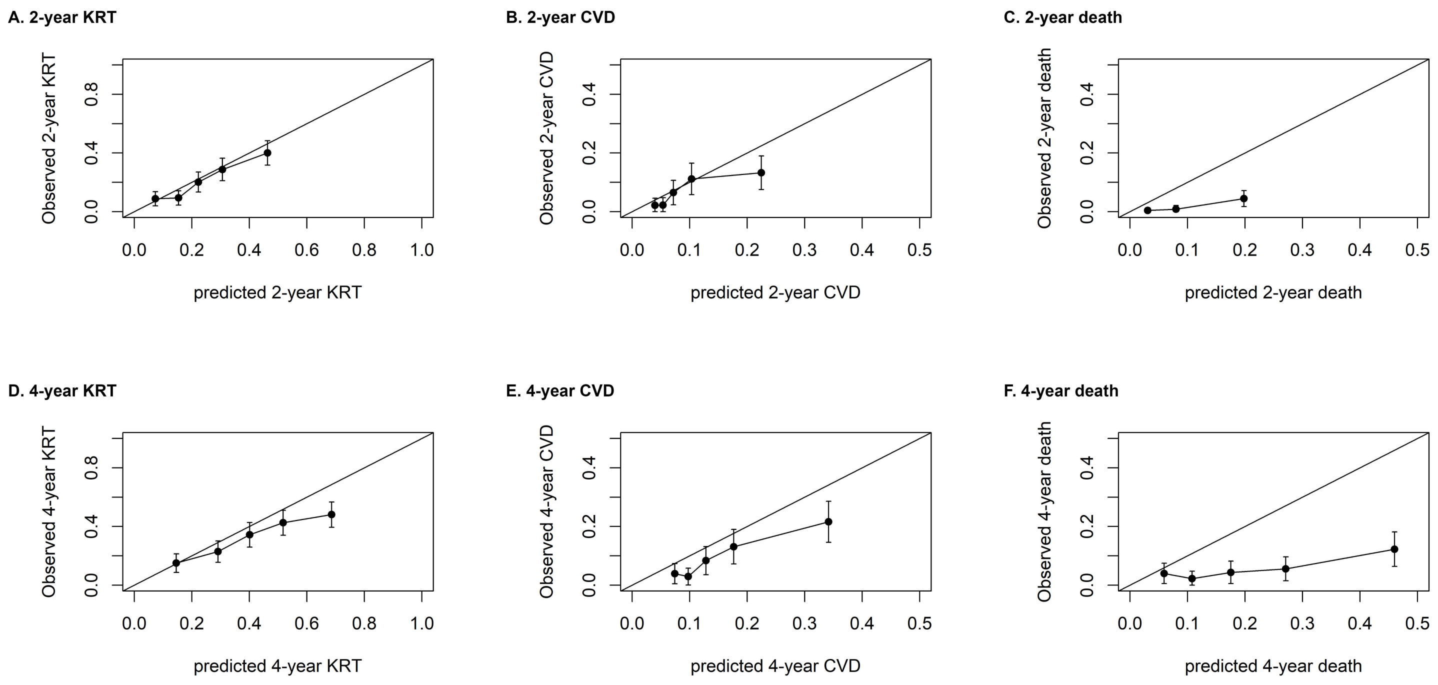

Figure S8. Calibration plots of the 2-year and 4-year Grams model among male patients (N=698). The predicted probability is shown on the x-axis and the observed probability is given on the y-axis. The dotted 45-degree line represents perfect agreement between predicted and observed probability. Error bars represent 95% confidence intervals. (A), the calibration plot for 2-year KRT; (B), the calibration plot for 2-year CVD; (C), the calibration plot for 2-year death; (D), the calibration for 4-year KRT; (E), the calibration plot for 4-year CVD; (F), the calibration plot for 4-year death. KRT, kidney replacement therapy; CVD, cardiovascular disease.

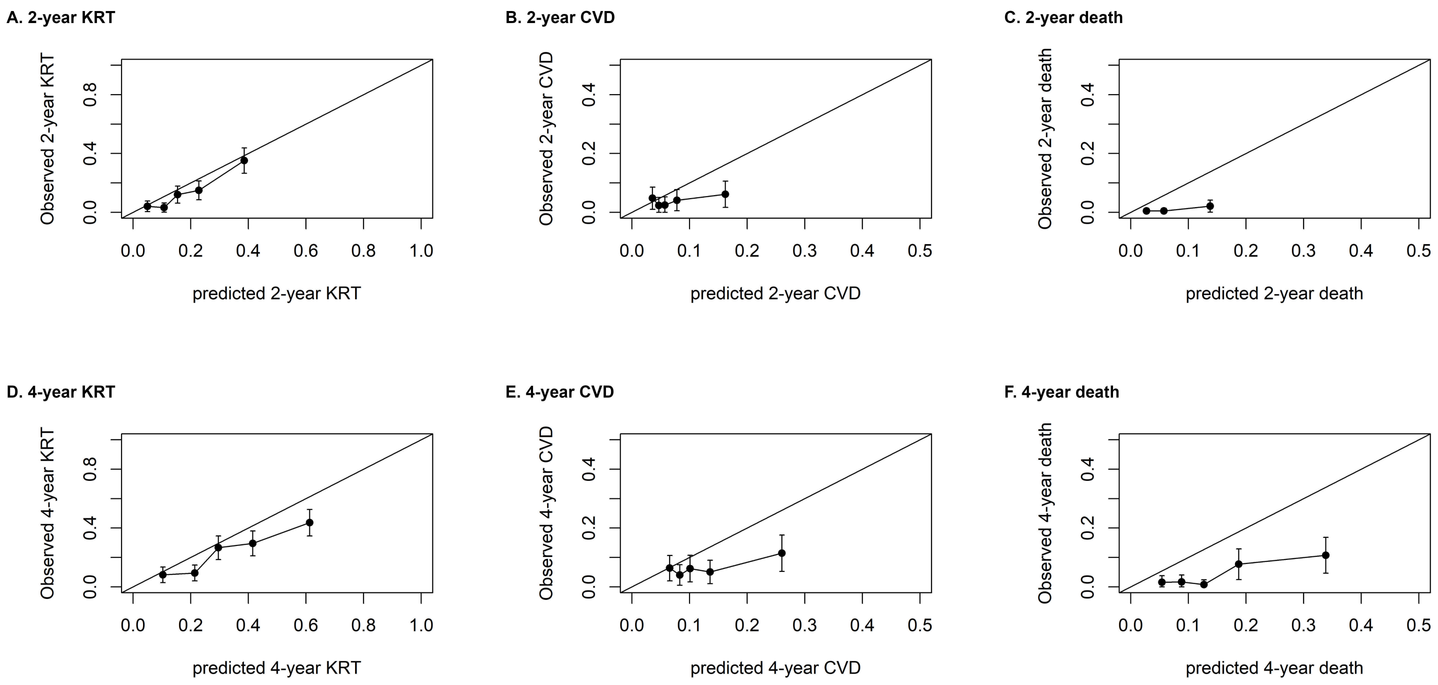

Figure S9. Calibration plots of the 2-year and 4-year Grams model among female patients (N=634). The predicted probability is shown on the x-axis and the observed probability is given on the y-axis. The dotted 45-degree line represents perfect agreement between predicted and observed probability. Error bars represent 95% confidence intervals. (A), the calibration plot for 2-year KRT; (B), the calibration plot for 2-year CVD; (C), the calibration plot for 2-year death; (D), the calibration for 4-year KRT; (E), the calibration plot for 4-year CVD; (F), the calibration plot for 4-year death. KRT, kidney replacement therapy; CVD, cardiovascular disease.

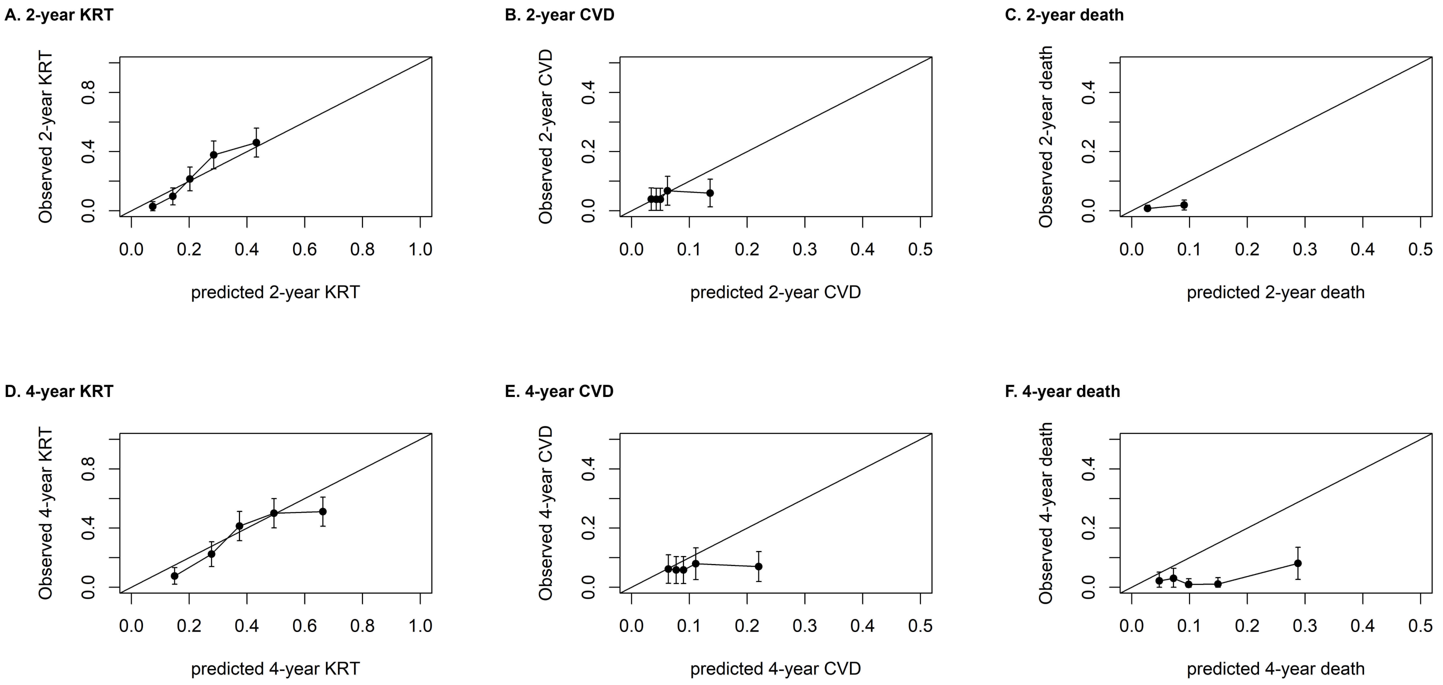

Figure S10. Calibration plots of the 2-year and 4-year Grams model among glomerulonephritis patients (N=519). The predicted probability is shown on the x-axis and the observed probability is given on the y-axis. The dotted 45-degree line represents perfect agreement between predicted and observed probability. Error bars represent 95% confidence intervals. (A), the calibration plot for 2-year KRT; (B), the calibration plot for 2-year CVD; (C), the calibration plot for 2-year death; (D), the calibration for 4-year KRT; (E), the calibration plot for 4-year CVD; (F), the calibration plot for 4-year death. KRT, kidney replacement therapy; CVD, cardiovascular disease.

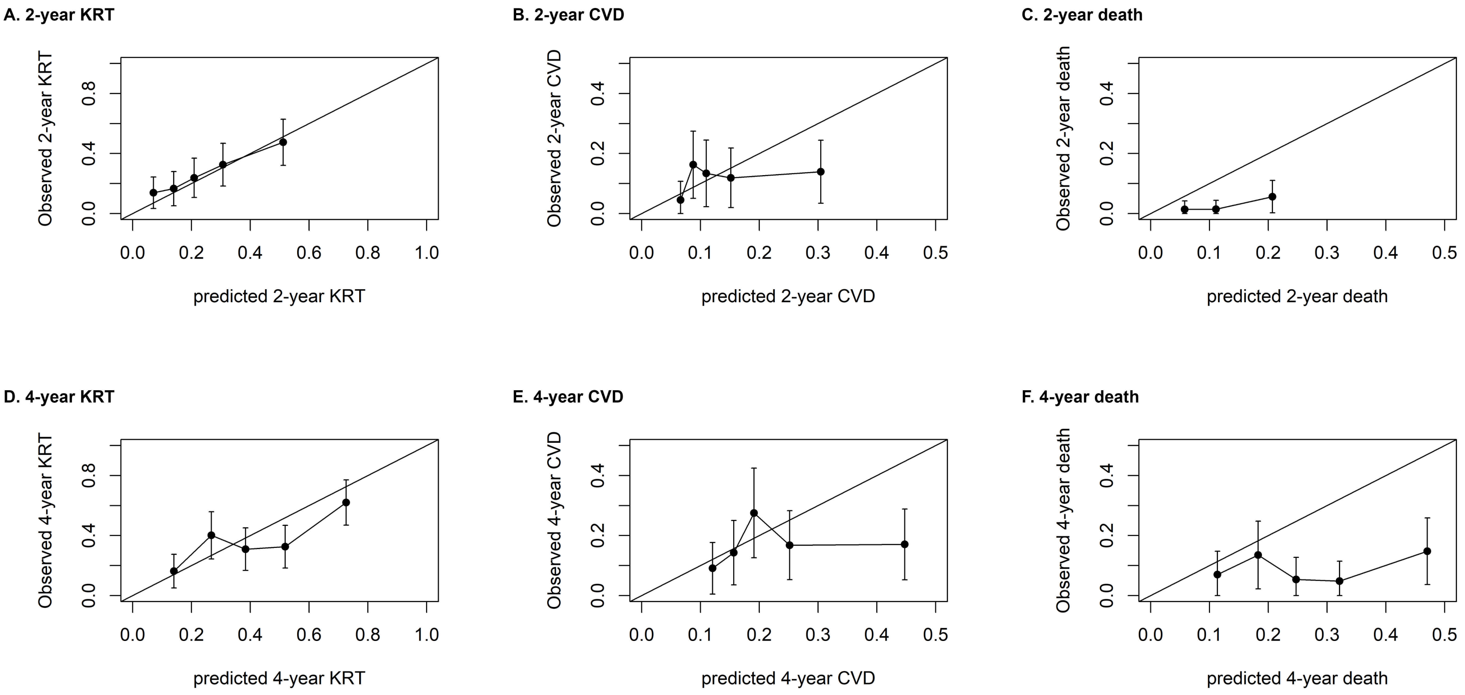

Figure S11. Calibration plots of the 2-year and 4-year Grams model among diabetic kidney disease patients (N=216). The predicted probability is shown on the x-axis and the observed probability is given on the y-axis. The dotted 45-degree line represents perfect agreement between predicted and observed probability. Error bars represent 95% confidence intervals. (A), the calibration plot for 2-year KRT; (B), the calibration plot for 2-year CVD; (C), the calibration plot for 2-year death; (D), the calibration for 4-year KRT; (E), the calibration plot for 4-year CVD; (F), the calibration plot for 4-year death. KRT, kidney replacement therapy; CVD, cardiovascular disease.

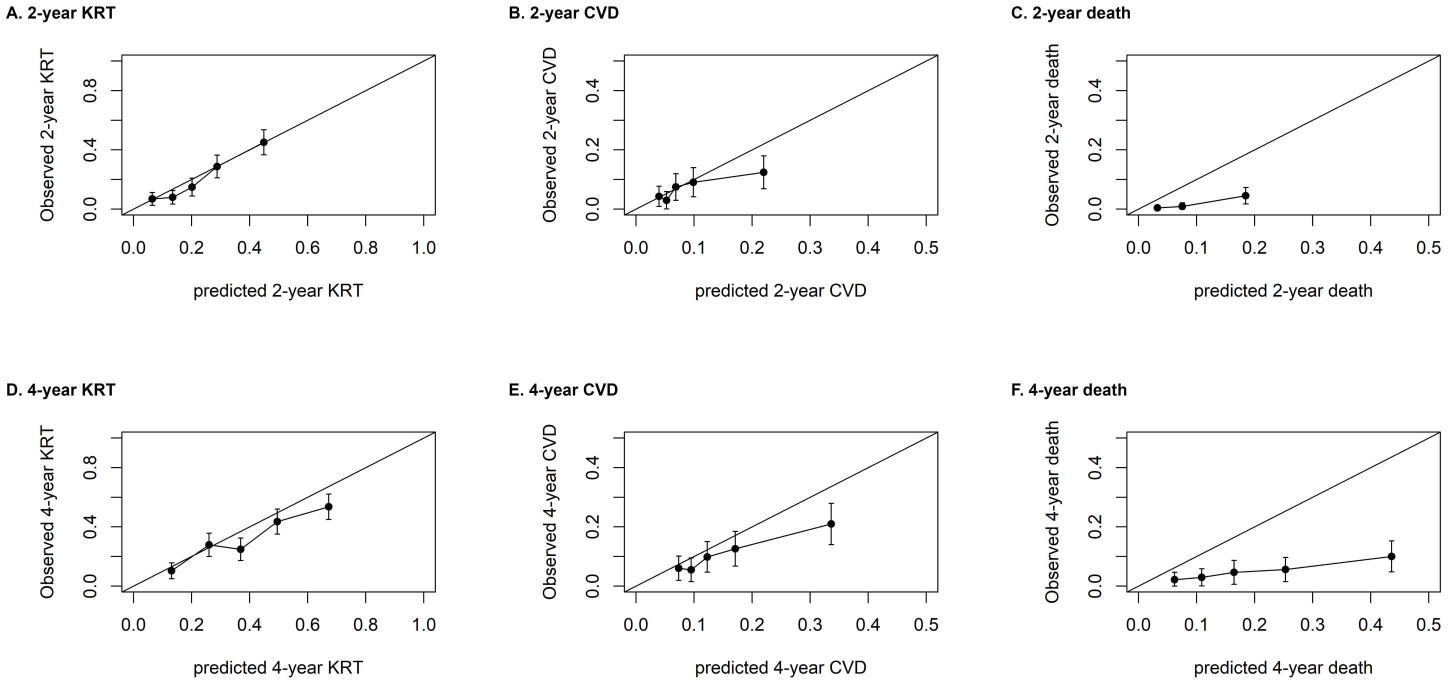

Figure S12. Calibration plots of the 2-year and 4-year Grams model when smoking status was defined as current smoking (N=691). The predicted probability is shown on the x-axis and the observed probability is given on the y-axis. The dotted 45-degree line represents perfect agreement between predicted and observed probability. The dots represent a quintile of the validation population, ranked by predicted probability. Error bars represent 95% confidence intervals. (A), the calibration plot for 2-year KRT; (B), the calibration plot for 2-year CVD; (C), the calibration plot for 2-year death; (D), the calibration for 4-year KRT; (E), the calibration plot for 4-year CVD; (F), the calibration plot for 4-year death. KRT, kidney replacement therapy; CVD, cardiovascular disease.

Table S1. Correction factors of intercept recalibration.

Linear predictors	Correction factors	
	2 years	4 years
KRT only vs No events	-0.28199622	-0.226136058
KRT after CVD vs No events	0.821519995	0.611787744
CVD only vs No events	-0.757791124	-0.531350532
CVD after KRT vs No events	-0.60491442	-0.665966035
Death only vs No events	-1.780831836	-1.231862682
Death after KRT vs No events	[a]	-1.745883118
Death after CVD vs No events	-1.22241513	-1.524201766
Death after KRT and CVD vs No events	-1.090824329	-2.165790528

Abbreviations: CVD, cardiovascular disease; KRT, kidney replacement therapy.

[a] In our cohort, there were no death after KRT within 2 years. But in order to make the correction factor computable, we set the occurrence rate of death after KRT to $1*10^{\wedge-13}$.

Table S2. Baseline and Follow-up characteristics of study participants

Characteristics [a]	C-STRIDE cohort(N=1333)
**Baseline**	
Age, median (IQR), y	54.0 (43.0-64.0)
Men	699 (52.4%)
Black	0 (0%)
History of CVD	174 (13.1%)
Diabetes mellitus	352 (26.4%)
SBP, median (IQR), mmHg	134 (122-146)
Smoking history	384 (28.8%)
eGFR, median (IQR), ml/min/1.73 m^2	22.3 (17.3-26.3)
uACR, median (IQR), mg/g	549.4 (158.7-1248.3)
**CKD etiology** [b]	
Diabetic kidney disease	218 (16.4%)
Glomerulonephritis	519 (38.9%)
Others	386 (29.0%)
**Medications**	
BP-lowing medications	693 (52.0%)
Hypoglycemic medications	217 (16.3%)
BP-lowing and hypoglycemic combination	184 (13.8%)
RAAS inhibitor	390 (29.3%)
**Geographic distribution**	
Northeast	37 (2.8%)
North	506 (38%)
central	277 (20.8%)
West	513 (38.5%)
**Outcomes**	
KRT event	386 (29.0%)
Time to KRT, median (IQR), y	3.8 (2.3-4.9)
Incidence rate of KRT (95% CI), per 1000 person-years	81.8 (74.2-90.1)
4-year cumulative incidence of KRT (95% CI)	28.3% (25.8%-30.8%)
CVD event	118 (8.9%)
Time to CVD, median (IQR), y	4.3 (3.4-5.3)
Incidence rate of CVD (95% CI), per 1000 person-years	21.5 (17.9-25.8)

4-year cumulative incidence of CVD (95% CI)	8.5% (7.0%-10.1%)
All-cause mortality	88 (6.6%)
Time to mortality, median (IQR), y	4.4 (3.6-5.4)
Incidence rate of mortality (95% CI), per 1000 person-years	15.6 (12.6-19.2)
4-year cumulative incidence of mortality (95% CI)	5.3% (4.0%-6.6%)

Abbreviations: C-STRIDE, Chinese Cohort Study of Chronic Kidney Disease; CVD, cardiovascular disease; SBP, systolic blood pressure; eGFR, estimated glomerular filtration rate; uACR, urine albumin-to-creatinine ratio; RAAS inhibitor, renin-angiotensin-aldosterone system inhibitor; KRT, kidney replacement therapy; IQR, interquartile range; CI, confidence interval.

[a] Values are median (IQR) or n (%).

[b] There were 210 participants with unregistered etiology, so the sum of diabetic kidney disease, glomerulonephritis, and other causes does not equal the total number of participants.

Table S3. Baseline and follow-up characteristics, compared to the development cohort.

Characteristics	C-STRIDE cohort (N=1333) [a]	CKD-PC development cohort (N=264296) [b]
**Baseline**		
Age (years)	54 (43-64)	72
Men (%)	699 (52.4%)	161221 (61.0%)
Black (%)	0 (0%)	24580 (9.3%)
History of CVD (%)	174 (13.1%)	119197 (45.1%)
Diabetes mellitus	352 (26.4%)	122105 (46.2%)
SBP (mmHg)	134 (122-146)	130
Smoke status (%)	384 (28.8%)	NR
eGFR (ml/min/1.73 m^2)	22.3 (17.3-26.3)	24
uACR (mg/g)	549.4 (158.7-1248.3)	85
**CKD etiology**[c]		
Diabetic kidney disease	218 (16.4%)	-
Glomerulonephritis	519 (38.9%)	-
Others	386 (29.0%)	-
**Outcomes**		
KRT (%)	386 (29.0%)	31541 (11.9%)
time to KRT (years)	3.8 (2.3-4.9)	3.5
CVD (%)	118 (8.9%)	70394 (26.6%)
time to CVD (years)	4.3 (3.4-5.3)	3.5
death (%)	88 (6.6%)	123985 (46.9%)
time to death(years)	4.4 (3.6-5.4)	3.5

Abbreviations: C-STRIDE, Chinese Cohort Study of Chronic Kidney Disease; CKD-PC, Chronic Kidney Disease Prognosis Consortium; CVD, cardiovascular disease; SBP, systolic blood pressure; eGFR, estimated glomerular filtration rate; uACR, urine albumin-to-creatinine ratio; KRT, kidney replacement therapy.

[a] Data are presented as median (interquartile range), or n (%).

[b] The means or medians of the overall CKD-PC cohort were calculated by taking the mean of all cohort-specific means or medians, hance standard deviation and interquartile range could not be provided.

[c] 210 participants did not register etiology, hance the numbers of DKD, GN and others did not sum to total participants.

Table S4. The proportions of all outcomes.

Outcomes	Proportions (N=1333)	
	**2 years**	**4 years**
KRT only	185 (13.9%)	287 (21.5%)
KRT after CVD	13 (1%)	19 (1.4%)
CVD only	33 (2.5%)	46 (3.5%)
CVD after KRT	9 (0.7%)	19 (1.4%)
Death only	12 (0.9%)	32 (2.4%)
Death after KRT	0 (0%)	14 (1.1%)
Death after CVD	6 (0.5%)	10 (0.8%)
Death after KRT and CVD	3 (0.2%)	5 (0.4%)

Abbreviations: CVD, cardiovascular disease; KRT, kidney replacement therapy

Table S5. Missing rates of predictors.

Predictors	Missing rates (N=1333)
Age	0
Sex	0
Race	0
History of CVD	0
Smoking status	202 (15.2%)
SBP	209 (15.7%)
Diabetes mellitus	0
eGFR	0
uACR	134 (10.1%)

Abbreviations: CVD, cardiovascular disease; SBP, systolic blood pressure; eGFR, estimated glomerular filtration rate; uACR, urine albumin-to-creatinine ratio.

Table S6. Comparison of baseline and follow-up characteristics of original data, complete cases, and multiple imputed datasets.

Characteristics[a]	Original data (N=1333)	Complete cases （N=909）	Multiple imputed dataset (N=1333)
**Baseline**			
Age (years)	54 (43-64)	53 (43-63)	54 (43-64)
Men (%)	699 (52.4%)	499 (54.9%)	699 (52.4%)
Black (%)	0 (0%)	0 (0%)	0 (0%)
History of CVD (%)	174 (13.1%)	135 (14.9%)	174 (13.0%)
Diabetes mellitus	352 (26.4%)	243 (26.7%)	352 (26.4%)
SBP (mmHg)	134 (122-146)	133(122-145)	134 (122-146)
Smoke status (%)	384 (28.8%)	314 (34.5%)	442 (33.1%)
eGFR (ml/min/1.73 m^2)	22.3 (17.3-26.3)	22.6 (18.1-26.4)	22.3 (17.3-26.3)
uACR (mg/g)	549.4 (158.7-1248.3)	530.4 (149.9-1263.1)	534.3 (152.7-1226.3)
**Outcomes**			
KRT (%)	386 (29.0%)	324 (35.6%)	386 (28.9%)
time to KRT (years)	3.8 (2.3-4.9)	3.8 (2.3-4.8)	3.8 (2.3-4.9)
CVD (%)	118 (8.9%)	95 (10.5%)	118 (8.8%)
time to CVD (years)	4.3 (3.4-5.3)	4.4 (3.5-5.3)	4.3 (3.4-5.3)
death (%)	88 (6.6%)	56 (6.2%)	88 (6.6%)
time to death (years)	4.4 (3.6-5.4)	4.5 (3.7-5.4)	4.4 (3.6-5.4)

Abbreviations: CVD, cardiovascular disease; SBP, systolic blood pressure; eGFR, estimated glomerular filtration rate; uACR, urine albumin-to-creatinine ratio; KRT, kidney replacement therapy.

[a] Data are presented as median (interquartile range), or n (%).

Table S7. Comparison of diagnostic performance for timing of KRT preparation based on different eGFR and Grams model risk thresholds

Decision criteria	Total referrals	TP	FP	TN	FN	Sensitivity (95% CI)	Specificity (95% CI)	PPV (95% CI)	NPV (95% CI)
eGFR< 30	1333	114	1219	0	0	1 (0.968, 1)	0 (0, 0.003)	0.086 (0.071, 0.102)	-
eGFR< 20	515	72	443	776	42	0.632 (0.536, 0.720)	0.637 (0.609, 0.664)	0.140 (0.111, 0.173)	0.949 (0.931, 0.963)
eGFR< 15	216	28	188	1031	86	0.246 (0.170, 0.335)	0.846 (0.824, 0.866)	0.130 (0.088, 0.182)	0.923 (0.906, 0.938)
2-year KRT risk > 0.2	481	89	392	827	25	0.781 (0.694, 0.853)	0.678 (0.651, 0.705)	0.185 (0.151, 0.223)	0.971 (0.957, 0.981)
2-year KRT risk > 0.3	222	52	170	1049	62	0.456 (0.363, 0.552)	0.861 (0.840, 0.880)	0.234 (0.180, 0.296)	0.944 (0.929, 0.957)
2-year KRT risk > 0.4	80	18	62	1157	96	0.158 (0.096, 0.238)	0.949 (0.935, 0.961)	0.225 (0.139, 0.332)	0.923 (0.907, 0.938)
2-year KRT risk > 0.5	27	7	20	1199	107	0.061 (0.025, 0.122)	0.984 (0.975, 0.990)	0.259 (0.111, 0.463)	0.918 (0.902, 0.932)
2-year KRT risk >0.5 or eGFR<15	221	31	190	1029	83	0.272 (0.193, 0.363)	0.844 (0.823, 0.864)	0.140 (0.097, 0.193)	0.925 (0.908, 0.940)

Abbreviations: KRT, kidney replacement therapy; eGFR, estimated glomerular filtration rate; TP, true positive; FP, false positive; TN, true negative; FN, false negative; PPV, positive predictive value; NPV, negative predictive value; CI, confidence interval.

Table S8. Discrimination of Grams model in all imputed datasets, 2(a) and 4(b) years.

a)

**Datasets**	**C index (95%CI)**		
	**KRT**	**CVD**	**Death**
Imputed dataset 1	0.716 (0.685-0.746)	0.657 (0.594-0.721)	0.711 (0.602-0.818)
Imputed dataset 2	0.715 (0.683-0.746)	0.659 (0.594-0.722)	0.711 (0.600-0.823)
Imputed dataset 3	0.715 (0.684-0.746)	0.655 (0.560-0.720)	0.711 (0.601-0.823)
Imputed dataset 4	0.712 (0.681-0.744)	0.657 (0.594-0.719)	0.711 (0.596-0.825)
Imputed dataset 5	0.717 (0.687-0.746)	0.659 (0.594-0.723)	0.711 (0.603-0.821)
Imputed dataset 6	0.719 (0.689-0.748)	0.658 (0.596-0.724)	0.712 (0.600-0.823)
Imputed dataset 7	0.718 (0.687-0.749)	0.658 (0.591-0.721)	0.711 (0.601-0.826)
Imputed dataset 8	0.716 (0.684-0.746)	0.656 (0.592-0.715)	0.712 (0.600-0.830)
Imputed dataset 9	0.718 (0.686-0.750)	0.658 (0.594-0.726)	0.712 (0.602-0.822)
Imputed dataset 10	0.713 (0.682-0.745)	0.656 (0.593-0.722)	0.712 (0.599-0.824)
Imputed dataset 11	0.713 (0.683-0.744)	0.658 (0.595-0.723)	0.712 (0.602-0.824)
Imputed dataset 12	0.714 (0.684-0.744)	0.657 (0.596-0.718)	0.712 (0.601-0.821)
Imputed dataset 13	0.720 (0.688-0.752)	0.656 (0.593-0.721)	0.712 (0.600-0.816)
Imputed dataset 14	0.713 (0.683-0.744)	0.658 (0.595-0.722)	0.712 (0.598-0.825)
Imputed dataset 15	0.717 (0.687-0.748)	0.655 (0.594-0.721)	0.711 (0.601-0.822)

b)

**Datasets**	**C index(95%CI)**		
	**KRT**	**CVD**	**death**
imputed dataset 1	0.686 (0.659-0.712)	0.656 (0.604-0.711)	0.682 (0.613-0.750)
imputed dataset 2	0.684 (0.656-0.712)	0.659 (0.606-0.712)	0.687 (0.621-0.758)
Imputed dataset 3	0.684 (0.656-0.711)	0.655 (0.600-0.708)	0.680 (0.614-0.748)

imputed dataset 4	0.682 (0.655-0.710)	0.658 (0.602-0.713)	0.684 (0.612-0.752)
imputed dataset 5	0.684 (0.657-0.710)	0.658 (0.602-0.712)	0.684 (0.617-0.750)
imputed dataset 6	0.685 (0.658-0.712)	0.659 (0.603-0.715)	0.683 (0.614-0.753)
imputed dataset 7	0.686 (0.661-0.714)	0.657 (0.603-0.710)	0.685 (0.617-0.757)
imputed dataset 8	0.685 (0.658-0.713)	0.655 (0.599-0.712)	0.687 (0.620-0.753)
imputed dataset 9	0.686 (0.660-0.712)	0.655 (0.598-0.709)	0.683 (0.615-0.750)
imputed dataset 10	0.682 (0.654-0.708)	0.659 (0.603-0.712)	0.682 (0.609-0.752)
imputed dataset 11	0.682 (0.656-0.709)	0.657 (0.605-0.710)	0.684 (0.614-0.752)
imputed dataset 12	0.684 (0.657-0.710)	0.657 (0.602-0.710)	0.681 (0.614-0.748)
imputed dataset 13	0.686 (0.659-0.714)	0.658 (0.601-0.710)	0.684 (0.616-0.753)
imputed dataset 14	0.681 (0.656-0.707)	0.659 (0.605-0.712)	0.684 (0.615-0.755)
imputed dataset 15	0.685 (0.657-0.712)	0.653 (0.599-0.709)	0.685 (0.616-0.750)

Abbreviations: C index, concordance index; CI, confidence interval; KRT, kidney replacement therapy; CVD, cardiovascular disease.

Table S9. Discrimination of the Grams model in complete cases (N=909).

	C index (95%CI)	
**Outcome**	**2-year model**	**4-year model**
KRT	0.737 (0.703-0.771)	0.702 (0.674-0.731)
CVD	0.652 (0.578-0.726)	0.661 (0.600-0.722)
Death	0.722 (0.596-0.848)	0.670 (0.582-0.758)

Abbreviations: C index, concordance index; CI, confidence interval; KRT, kidney replacement therapy; CVD, cardiovascular disease.

Table S10. Discrimination of the Grams model when smoking status was defined as current smoking (N=691).

	C index (95%CI)	
**Outcome**	**2-year model**	**4-year model**
KRT	0.721 (0.683-0.760)	0.689 (0.656-0.725)
CVD	0.653 (0.581-0.723)	0.660 (0.600-0.720)
Death	0.729 (0.593-0.863)	0.669 (0.571-0.761)

Abbreviations: C index, concordance index; CI, confidence interval; KRT, kidney replacement therapy; CVD, cardiovascular disease.

References

1. Grams ME, Sang Y, Ballew SH, et al. Predicting timing of clinical outcomes in patients with chronic kidney disease and severely decreased glomerular filtration rate. *Kidney Int.* Jun 2018;93(6):1442-1451.
2. Ramspek CL, Boekee R, Evans M, et al. Predicting Kidney Failure, Cardiovascular Disease and Death in Advanced CKD Patients. *Kidney Int Rep.* Oct 2022;7(10):2230-2241.
