# Supplementary figures and images for: Predicting kidney replacement therapy, cardiovascular disease and all-cause mortality in advanced chronic kidney disease among the Chinese population
Source: Ren Fail. 2025 Sep 9;47(1):2556301. doi: 10.1080/0886022X.2025.2556301 (PMC12422041; doi:10.1080/0886022X.2025.2556301)

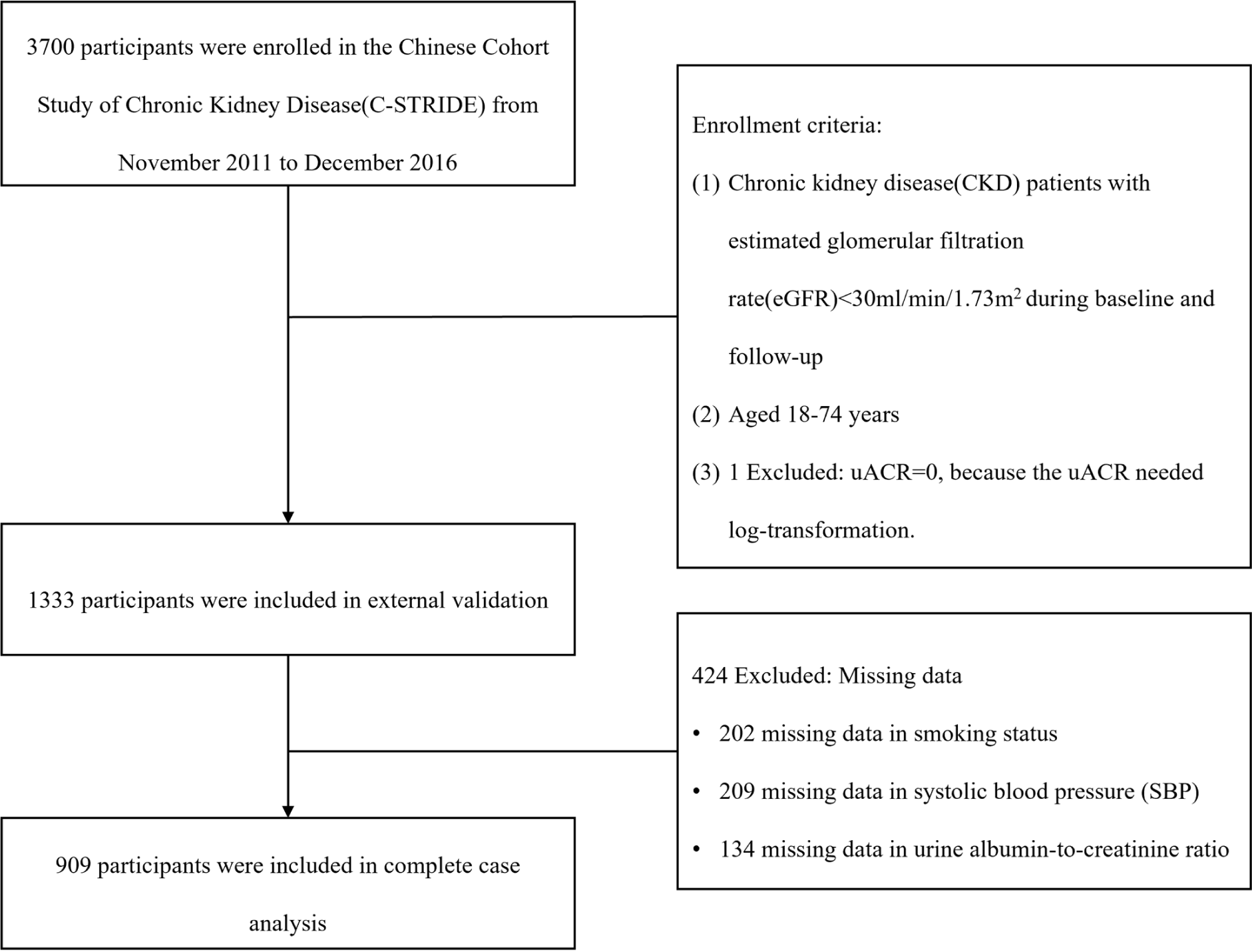

Supplement: supplemental_figures.zip [file IRNF_A_2556301_SM3109.zip › figure S1.tif]

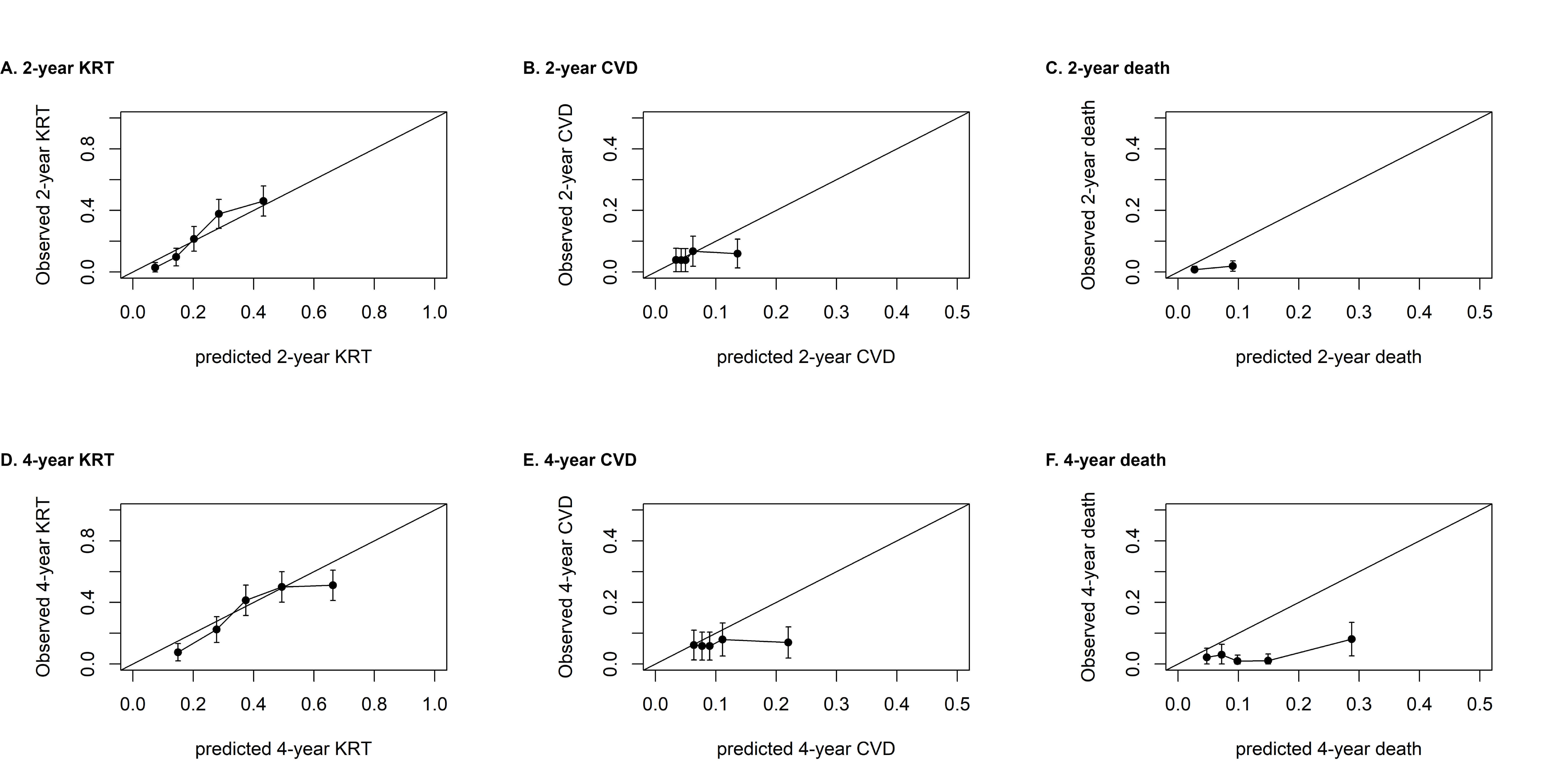

Supplement: supplemental_figures.zip [file IRNF_A_2556301_SM3109.zip › figure S10.tif]

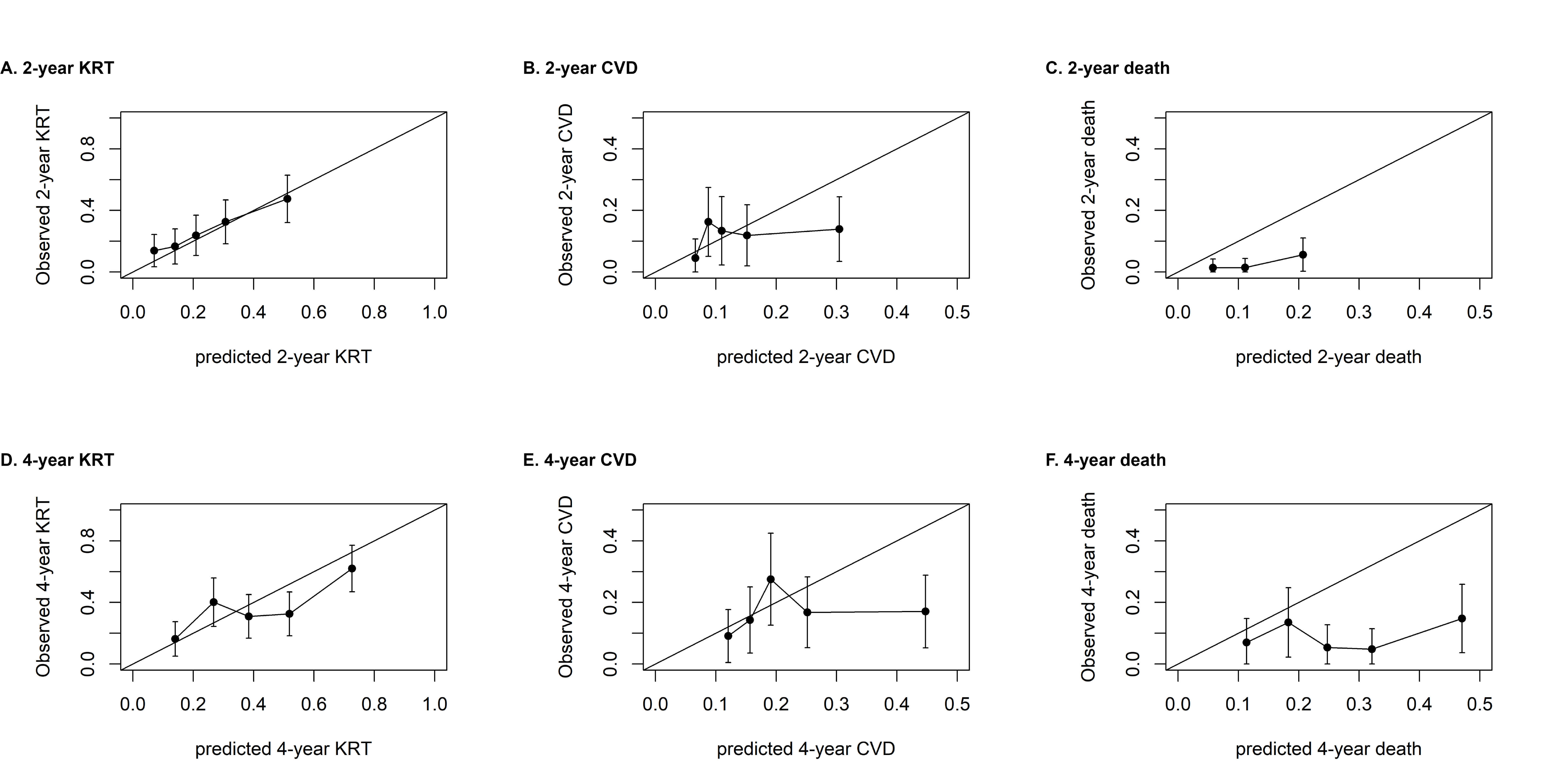

Supplement: supplemental_figures.zip [file IRNF_A_2556301_SM3109.zip › figure S11.tif]

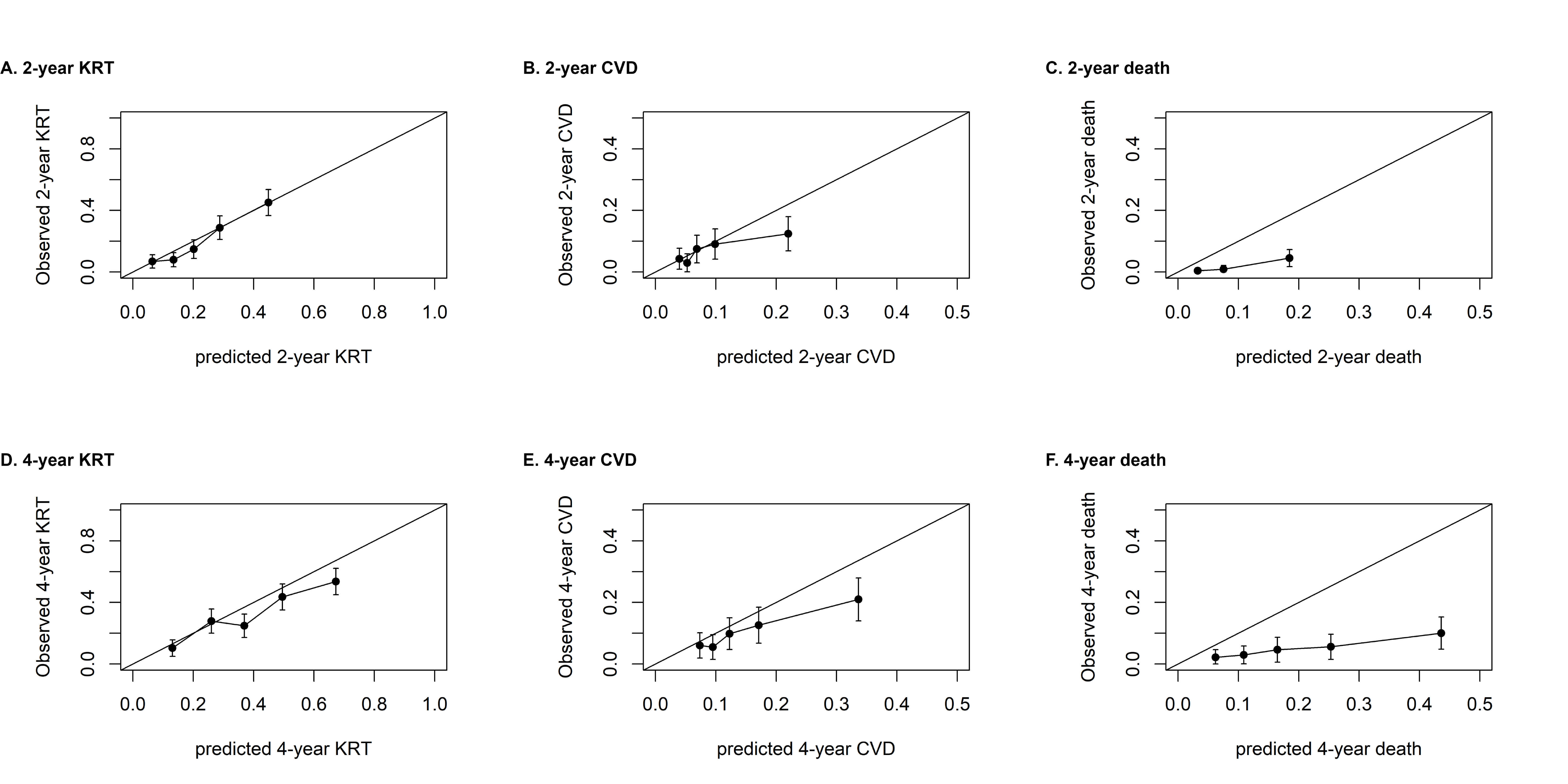

Supplement: supplemental_figures.zip [file IRNF_A_2556301_SM3109.zip › figure S12.tif]

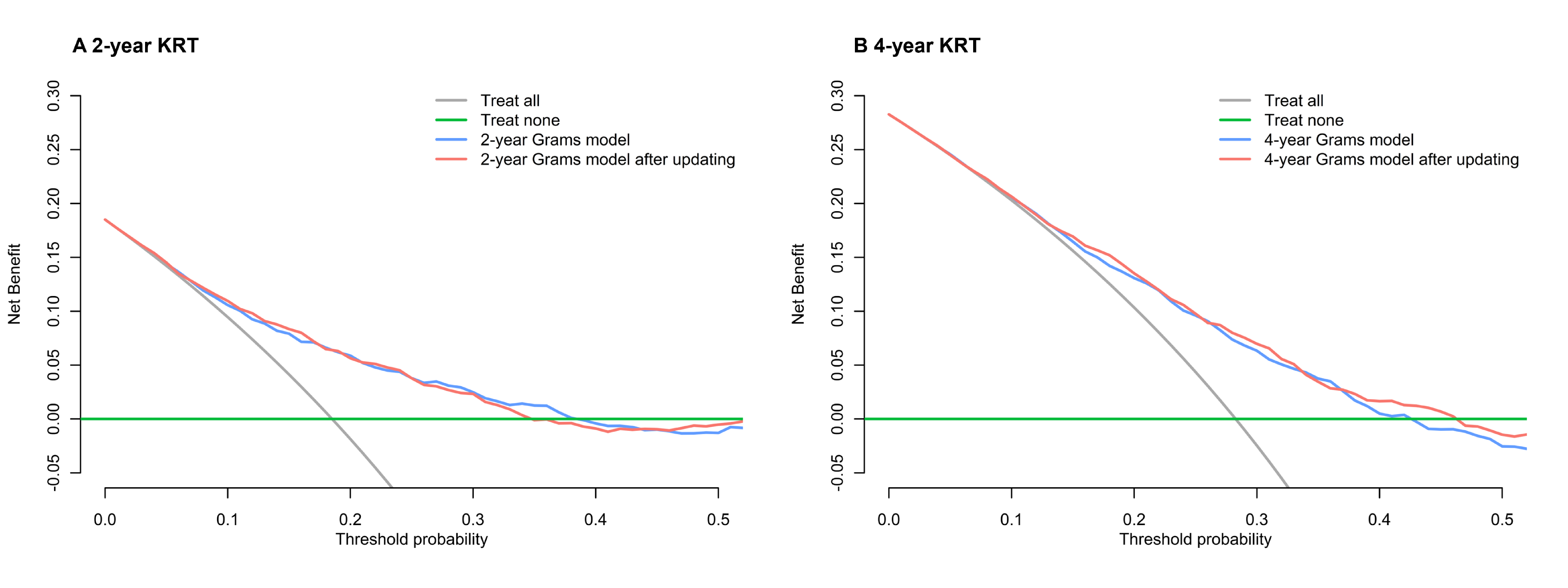

Supplement: supplemental_figures.zip [file IRNF_A_2556301_SM3109.zip › figure S2.tif]

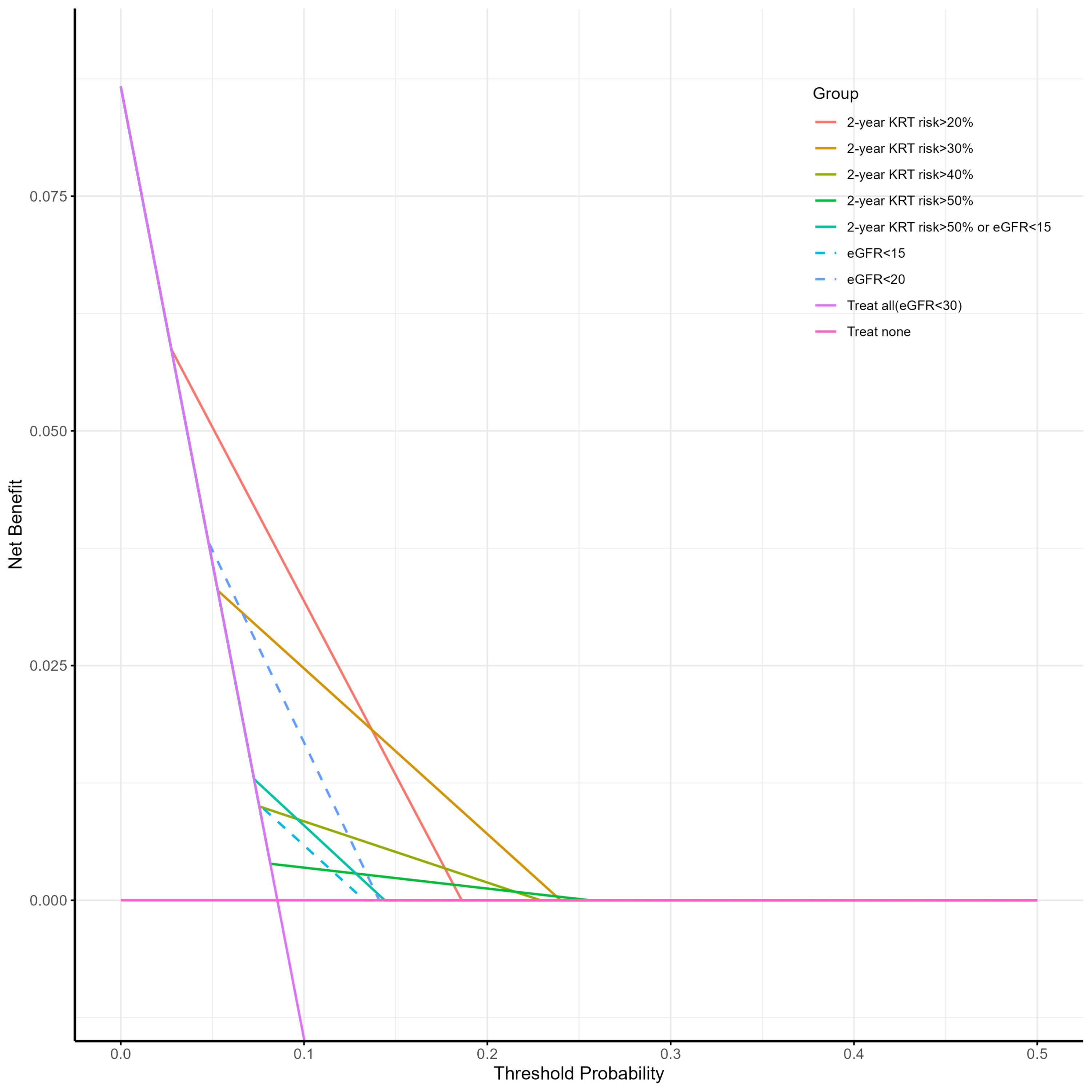

Supplement: supplemental_figures.zip [file IRNF_A_2556301_SM3109.zip › figure S3.tif]

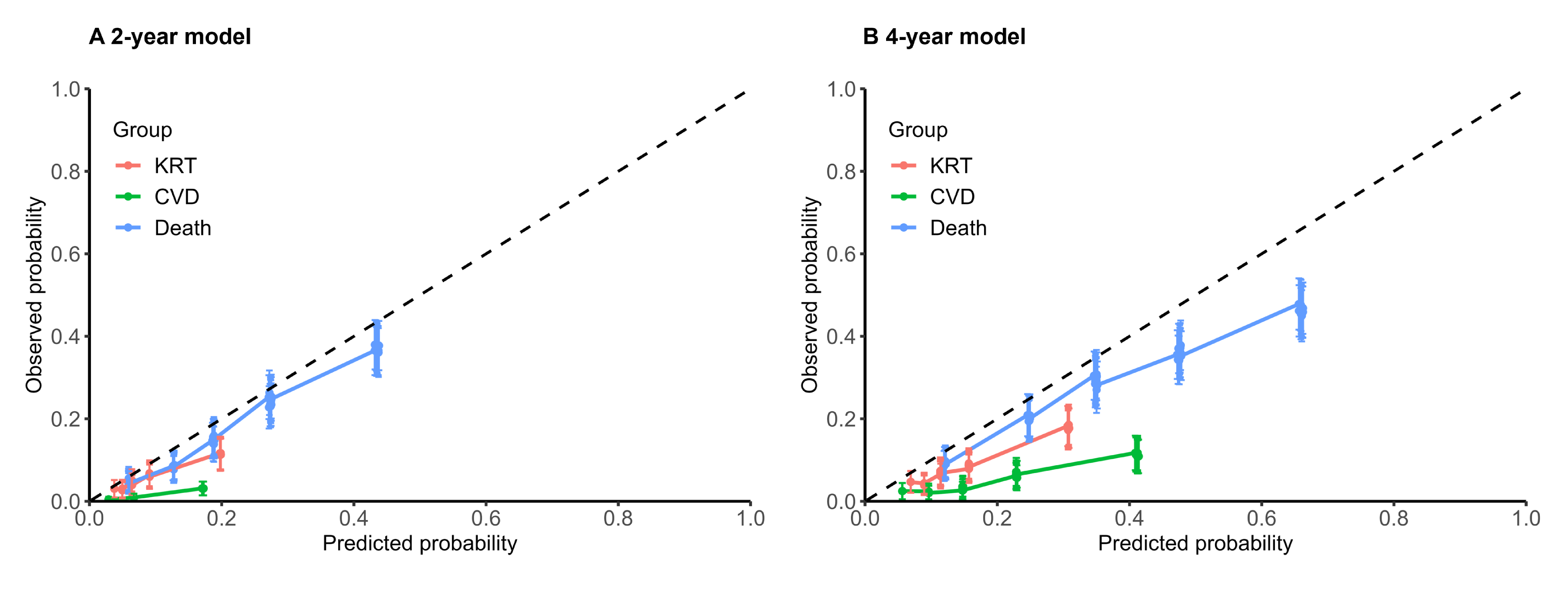

Supplement: supplemental_figures.zip [file IRNF_A_2556301_SM3109.zip › figure S4.tif]

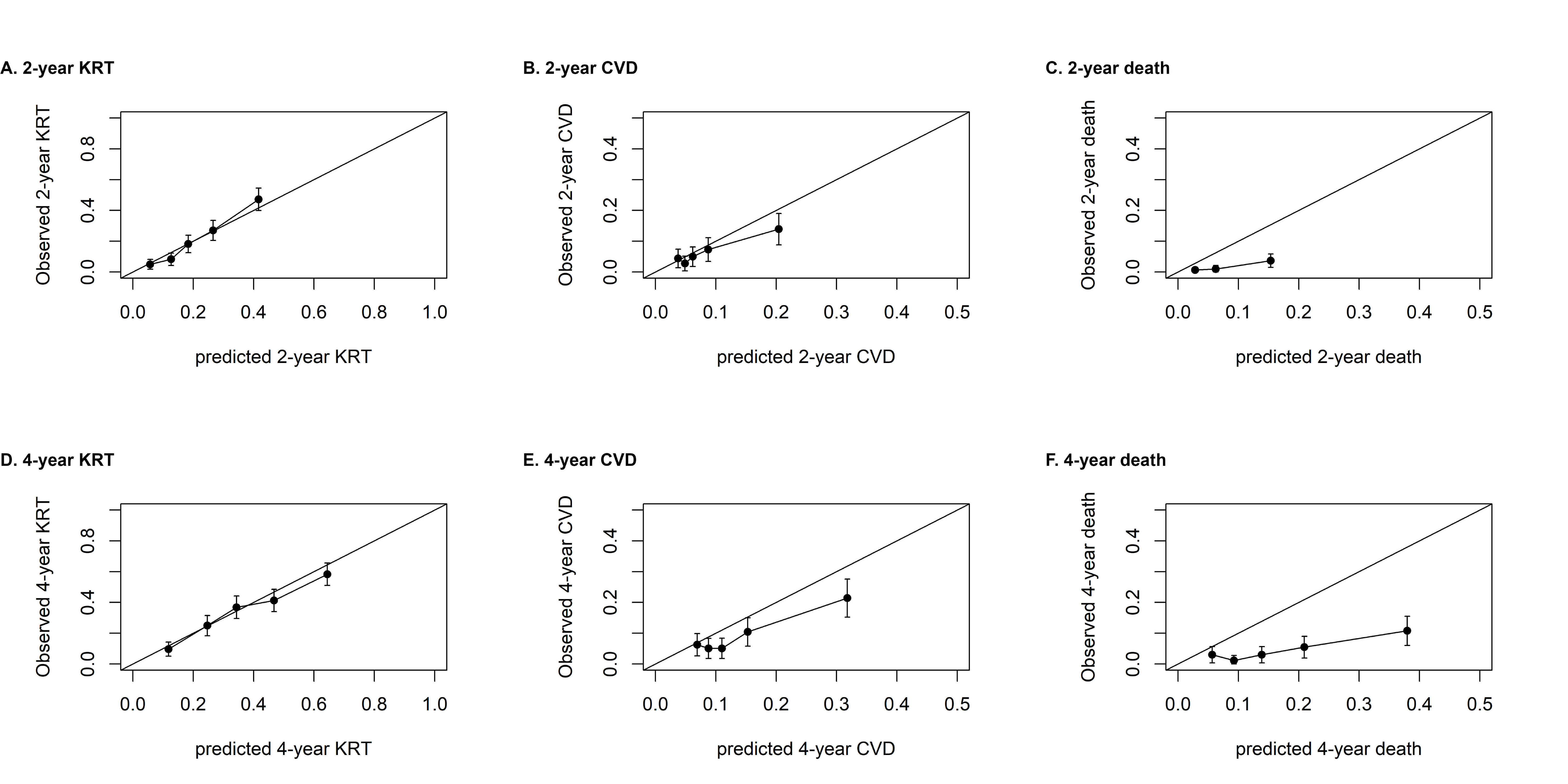

Supplement: supplemental_figures.zip [file IRNF_A_2556301_SM3109.zip › figure S5.tif]

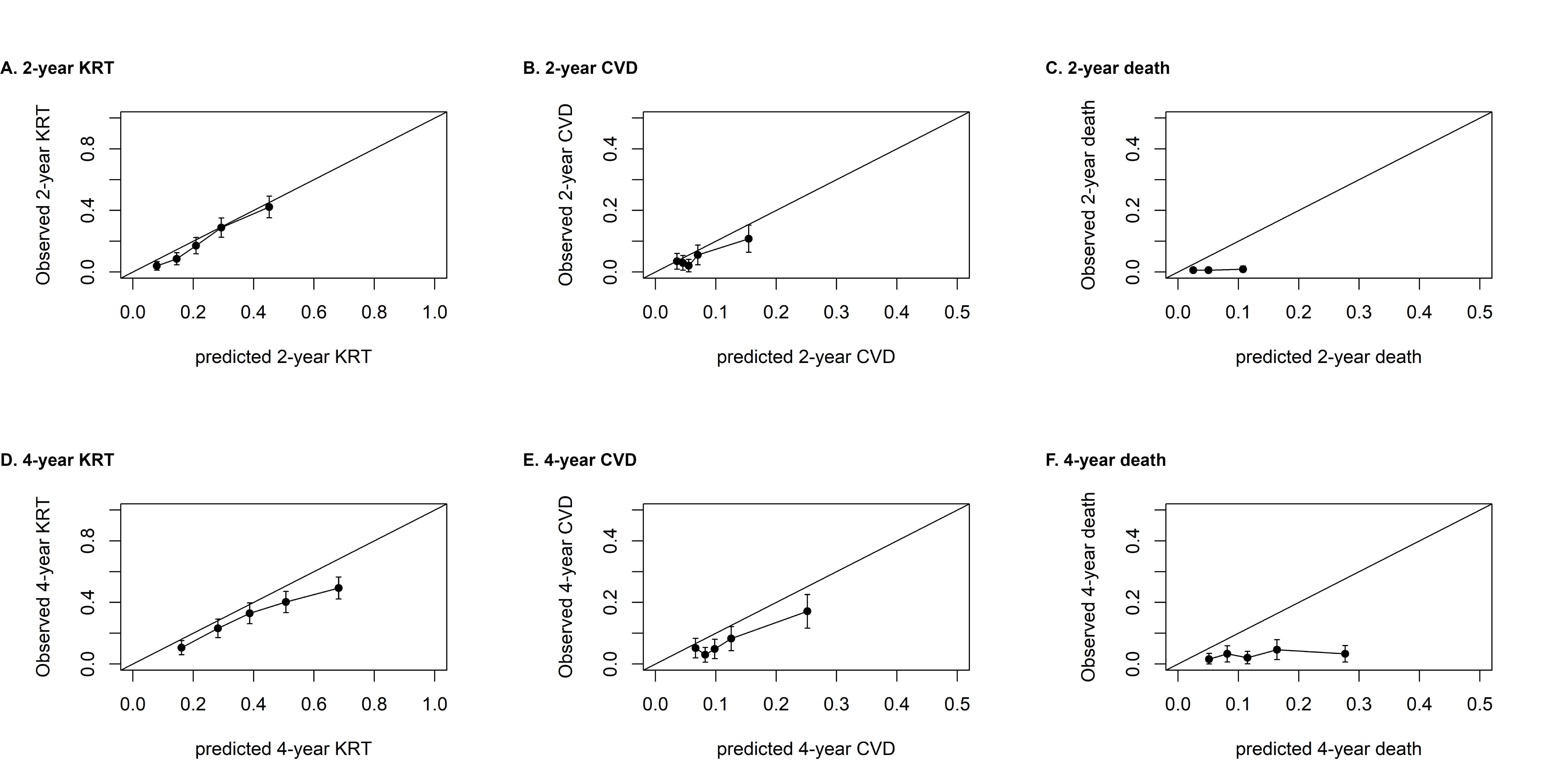

Supplement: supplemental_figures.zip [file IRNF_A_2556301_SM3109.zip › figure S6.tif]

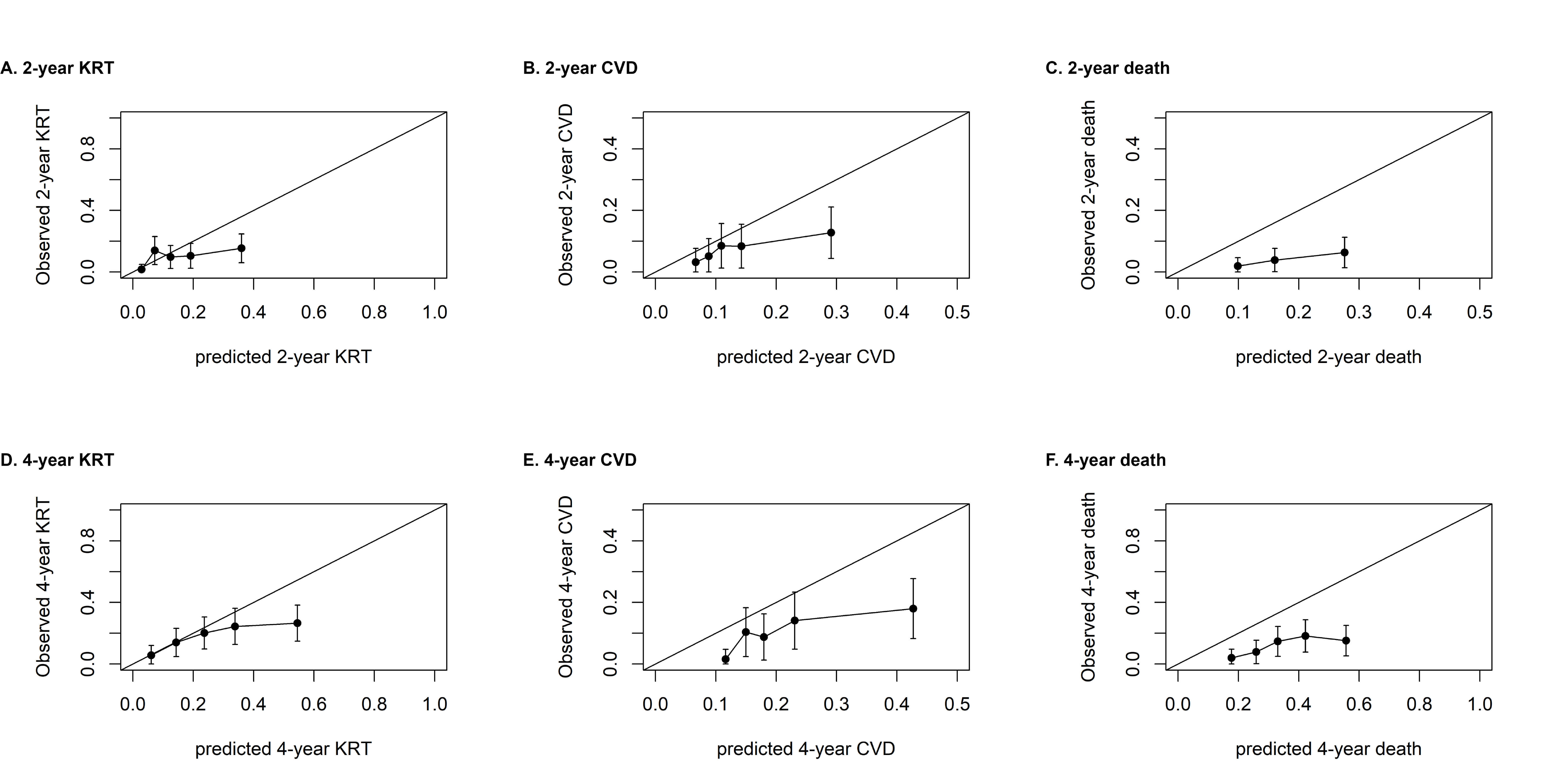

Supplement: supplemental_figures.zip [file IRNF_A_2556301_SM3109.zip › figure S7.tif]

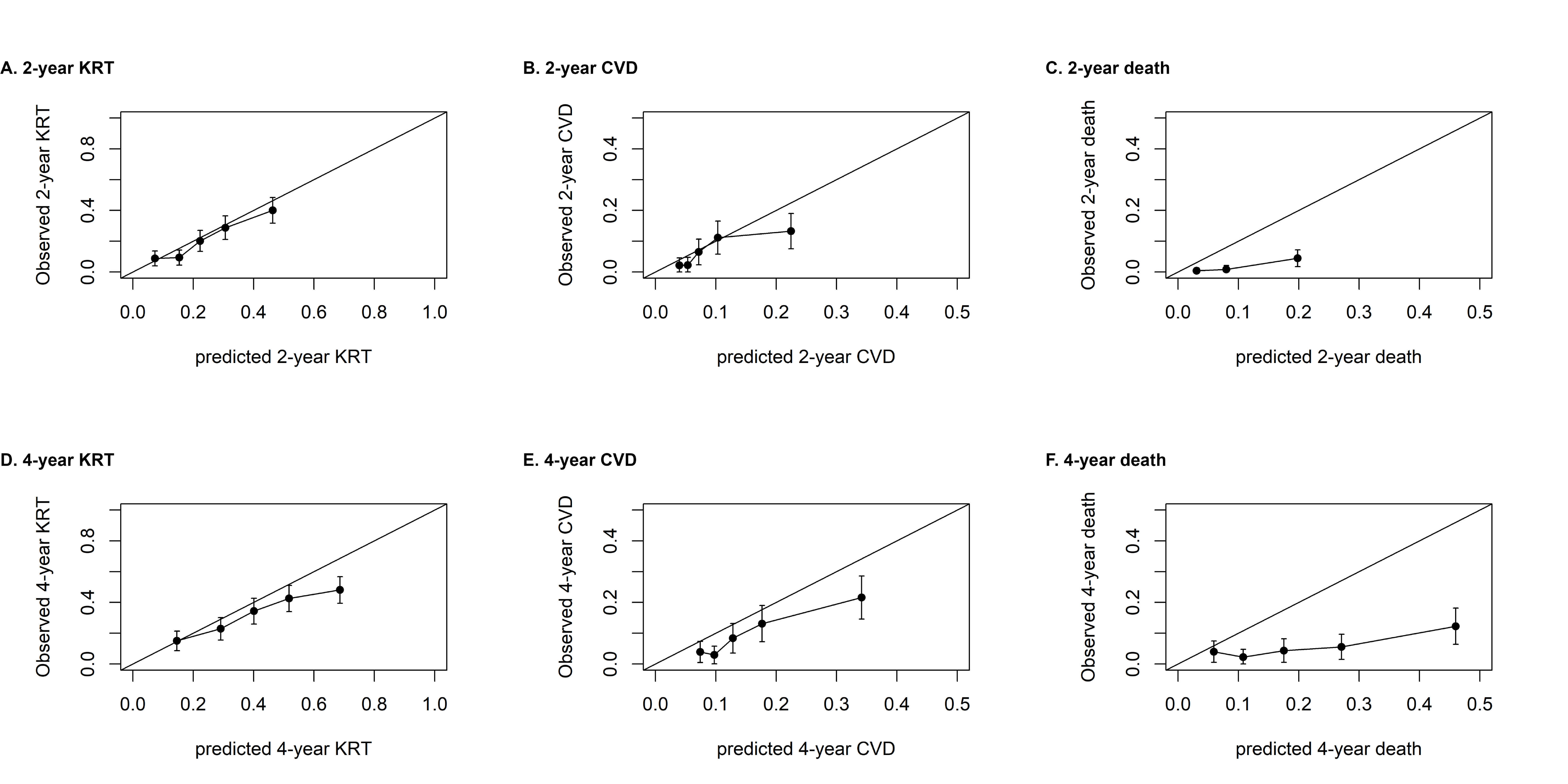

Supplement: supplemental_figures.zip [file IRNF_A_2556301_SM3109.zip › figure S8.tif]

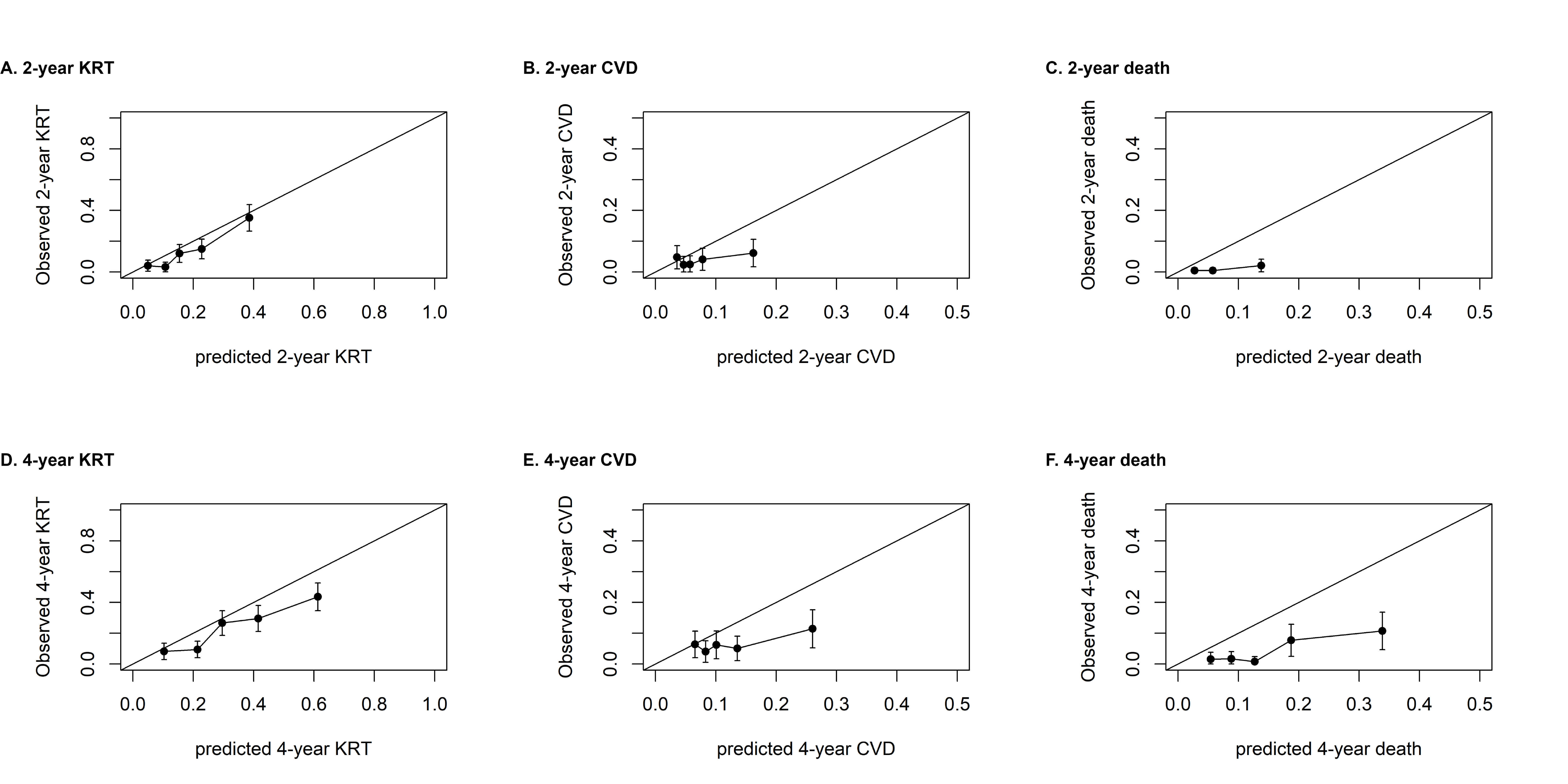

Supplement: supplemental_figures.zip [file IRNF_A_2556301_SM3109.zip › figure S9.tif]
